# Supplementary material for: A growth selection system for the directed evolution of amine-forming or converting enzymes
Source: Nat Commun. 2022 Dec 3;13:7458. doi: 10.1038/s41467-022-35228-y (PMC9718777; doi:10.1038/s41467-022-35228-y)
Supplement: Supplementary file 1 — Supplementary Information [file 41467_2022_35228_MOESM1_ESM.pdf]

Supplementary Information for:

A growth selection system for the directed evolution of amine-forming or converting enzymes

Shuke Wu[1,2,6]*, Chao Xiang[1,6], Yi Zhou[2], Mohammad Saiful Hasan Khan[1], Weidong Liu[1,3], Christian G. Feiler[4], Ren Wei[1], Gert Weber[4], Matthias Höhne[5] and Uwe T. Bornscheuer[1]*

[1] Department of Biotechnology and Enzyme Catalysis, Institute of Biochemistry, University of Greifswald, Felix Hausdorff-Str. 4, D-17489 Greifswald, Germany

[2] State Key Laboratory of Agricultural Microbiology, College of Life Science and Technology, Huazhong Agricultural University, No. 1 Shizishan Street, Wuhan 430070, P. R. China

[3] Industrial Enzymes National Engineering Laboratory, Tianjin Institute of Industrial Biotechnology, Chinese Academy of Sciences, Tianjin 300308, P. R. China

[4] Macromolecular Crystallography, Helmholtz-Zentrum Berlin für Materialien und Energie, Albert-Einstein-Straße 15, 12489 Berlin, Germany

[5] Protein Biochemistry, Institute of Biochemistry, University of Greifswald, Felix Hausdorff-Str. 4, D-17489 Greifswald, Germany

[6] These authors contributed equally: Shuke Wu, Chao Xiang

*e-mail: shukewu@mail.hzau.edu.cn; uwe.bornscheuer@uni-greifswald.de

Table of Contents

Supplementary Figures 3

Supplementary Tables 28

Supplementary Methods 31

Chemicals, Materials, and Software 31

Culture Media 32

DNA Sequences 32

General Method for Molecular Cloning, Transformation, and Engineering of Vectors 35

Genetic Engineering of AtTA 36

Genetic Engineering of CHAO 37

Genetic Engineering of PcPAL 38

Genetic Engineering of Assisting Plasmids 39

Genome Engineering of *E. coli* BL21(DE3) Δ*tyrB* 39

Expression of Enzymes and Preparation of Whole-cell Catalysts 40

Purification of Enzymes 40

Activity Assays 41

Analytical Methods 41

Crystallization, Data Collection, Structure Determination of AtTA(RHC) 42

Homology Modeling and Docking Experiment 42

Preparative Scale Syntheses 44

Supplementary References 45

Supplementary Figures

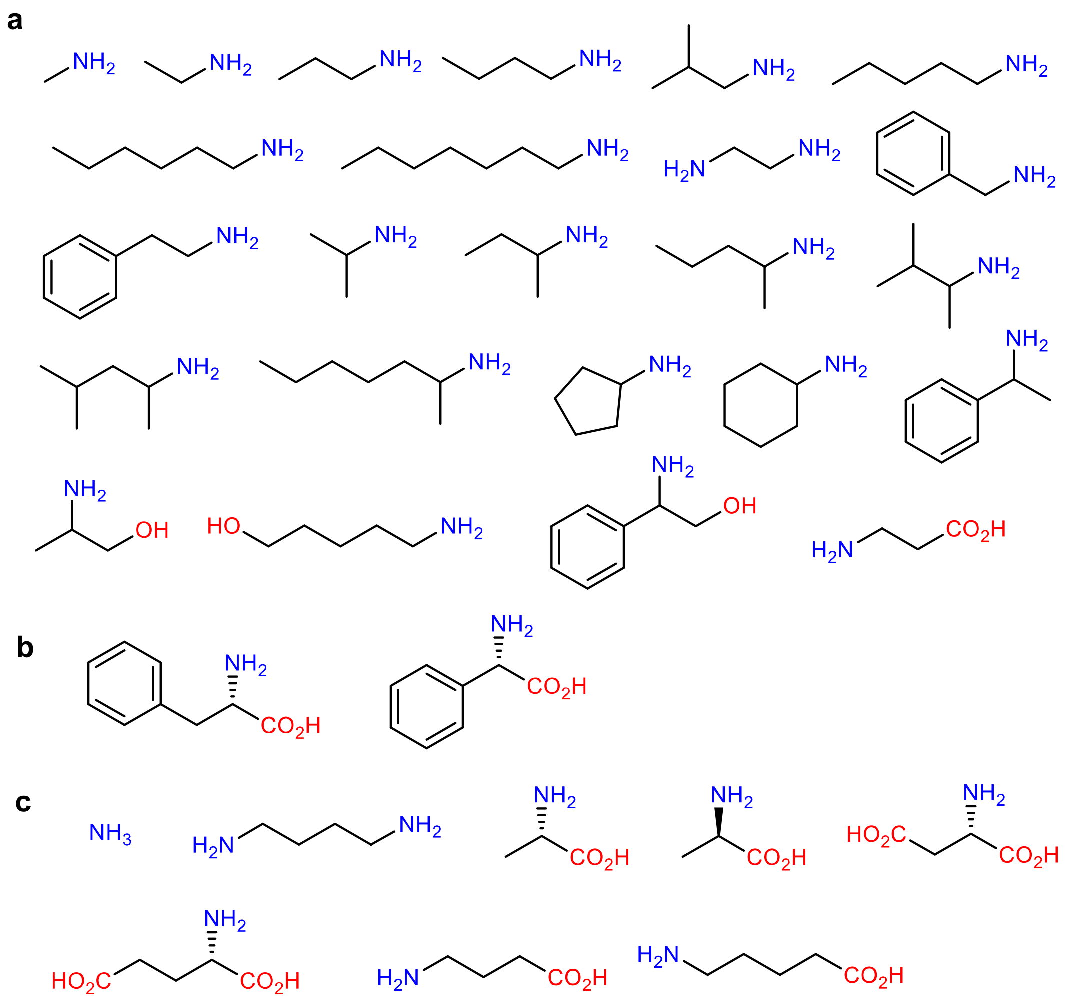

Supplementary Figure 1. Growth of *E. coli* BL21(DE3) with different amines and amino acids as the only nitrogen source. **a** No growth on these amines (OD_{600} < 0.1 in 72 h). **b** Moderate growth on these amino acids (0.1 < OD_{600} < 0.5 in 72 h). **c** Growth on these amines and amino acids (OD_{600} > 0.5 in 72 h). Conditions: An aliquot (20 µl) of an overnight culture of *E. coli* BL21(DE3) containing an empty plasmid in LB medium was inoculated into M9 medium (2 ml) with glucose (4 g l^{-1}) and the amine or amino acid (10 mM) as the sole nitrogen source and incubated at 30 °C, 200 rpm for 72 h. The growth test did not consider the toxicity of the amines or amino acids.

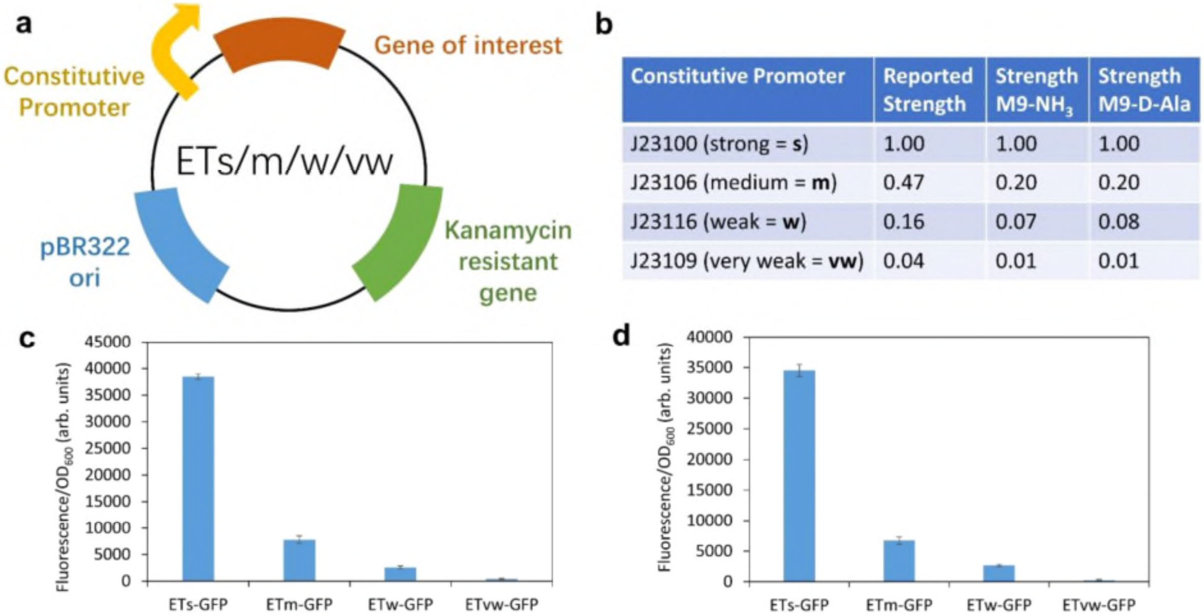

Supplementary Figure 2. Vectors and constitutive promoters used for growth selection in this study. **a** The ETs/m/w/vw plasmids used in the selection consist of a pBR322 ori, a kanamycin resistance gene, and the gene of interest under the control of a constitutive promoter (strong, medium, weak, and very weak strength). The details for the genetic engineering of the plasmids are provided in the method part. **b** The four constitutive promoters used in this study. The reported strength is according to a previous study (https://parts.igem.org/Part:BBa_J23100). The strength in M9-NH_3 and M9-D-Ala was measured by using the fluorescence of superfolder GFP. **c** The strength of the promoters was measured by the GFP fluorescence of the cells in M9-NH_3 medium. *E. coli* BL21(DE3) cells containing ETs/m/w/vw-GFP plasmid were inoculated (1%) in M9 medium (1 ml) with NH_3 (10 mM) as the sole nitrogen source, and incubated in a deep 96-well plate at 30 °C and 800 rpm for 24 h. **d** The strength of the promoters was measured by the GFP fluorescence of the cells in M9-D-Ala medium. *E. coli* BL21(DE3) cells containing ETs/m/w/vw-GFP plasmid were inoculated (1%) in M9 medium (1 ml) with D-Ala (10 mM) as the sole nitrogen source, and incubated in a deep 96-well plate at 30 °C and 800 rpm for 24 h. Fluorescence and OD_{600} data were measured at the end of the cultivation. Source data are provided as a Source Data file. Data in **c** and **d** are mean values of quadruplicate experiments with error bars indicating the s. d. (n = 4).

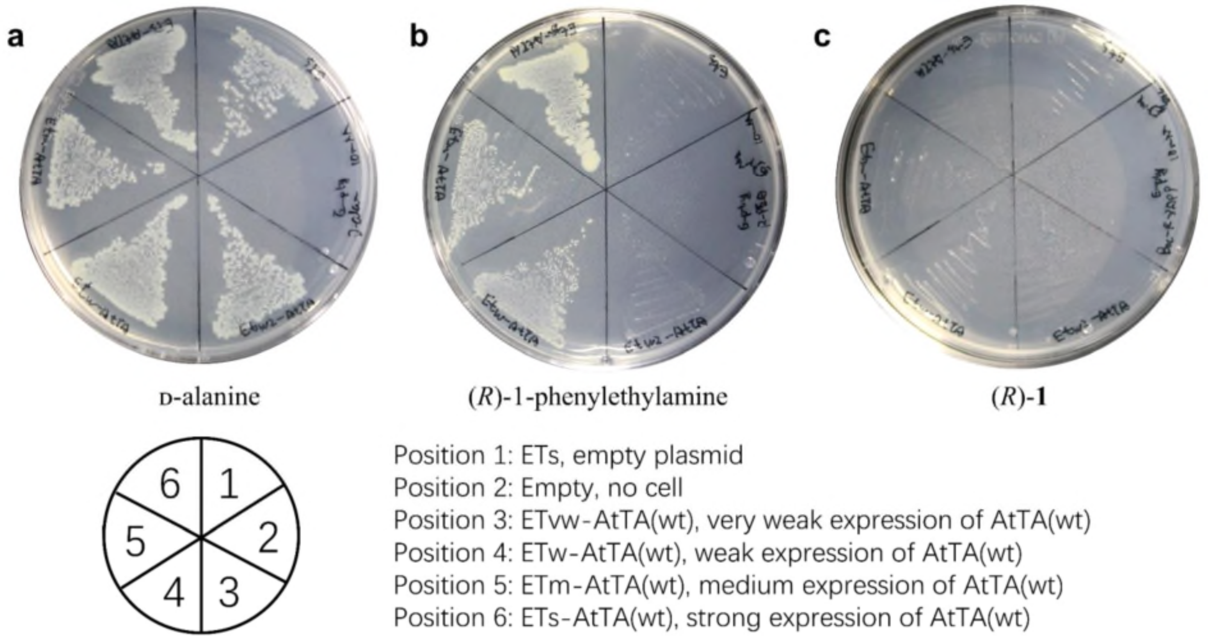

Supplementary Figure 3. Growth of *E. coli* cells expressing AtTA(wt) on M9 agar plates with different nitrogen sources. a Growth of the cells on an M9 agar plate with D-alanine (10 mM) as the only nitrogen source. **b** Growth of the cells on an M9 agar plate with (*R*)-1-phenylethylamine (10 mM) as the only nitrogen source. **c** Growth of the cells on an M9 agar plate with (*R*)-**1** (10 mM) as the only nitrogen source. *E. coli* BL21(DE3) cells containing ETs/m/w/vw-AtTA(wt) or ETs plasmid were streaked on the M9 agar plates with different nitrogen sources and incubated at 30 °C for 72 h then photos were taken.

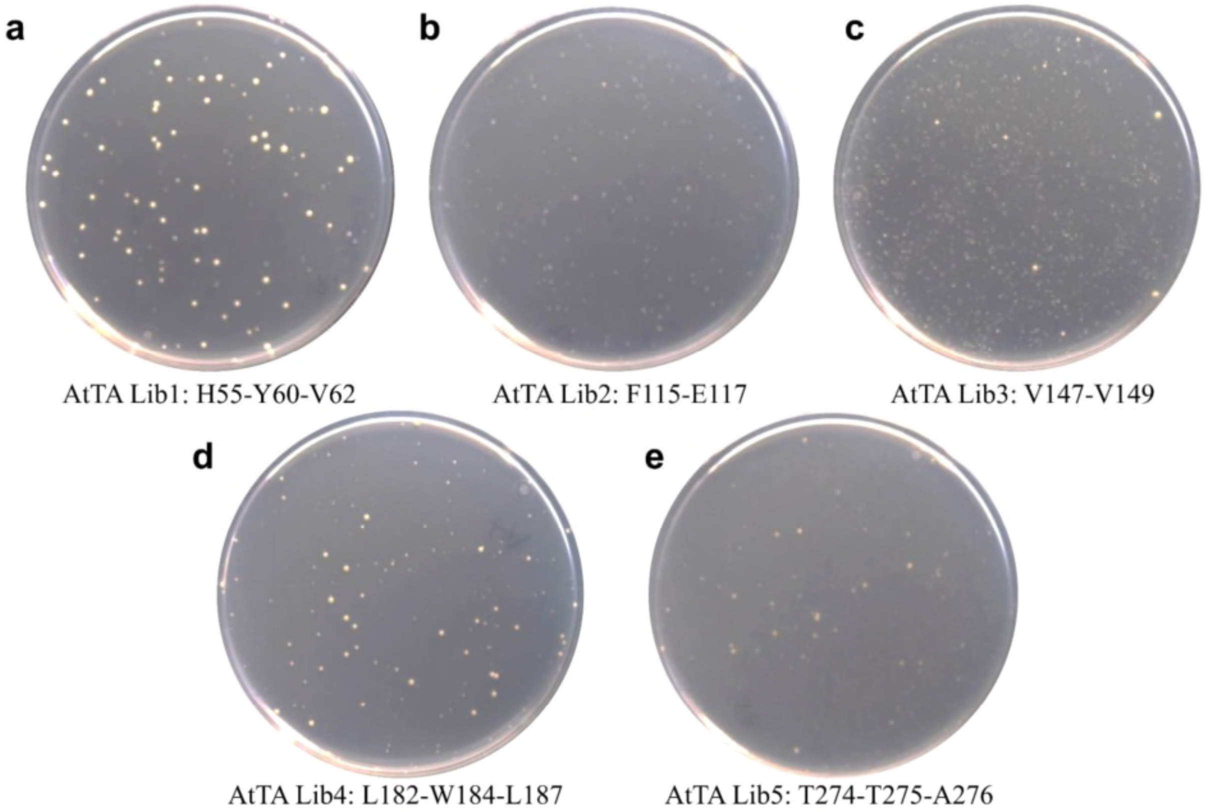

Supplementary Figure 4. Growth of *E. coli* cells containing ETs-AtTA libraries (strong expression levels) on M9 agar plates with (*R*)-1 (10 mM) as the sole nitrogen source. a Growth of *E. coli* cells containing the ETs-AtTA-Lib1 (H55-Y60-V62). **b** Growth of *E. coli* cells containing the ETs-AtTA-Lib2 (F115-E117). **c** Growth of *E. coli* cells containing the ETs-AtTA-Lib3 (V147-V149). **d** Growth of *E. coli* cells containing the ETs-AtTA-Lib4 (L182-W184-L187). **e** Growth of *E. coli* cells containing the ETs-AtTA-Lib5 (T274-T275-A276). Due to the close of the university during the outbreak of COVID-19 (no incubator was available), the plates were kept at room temperature (~15 °C) for 3 weeks and photos were taken then.

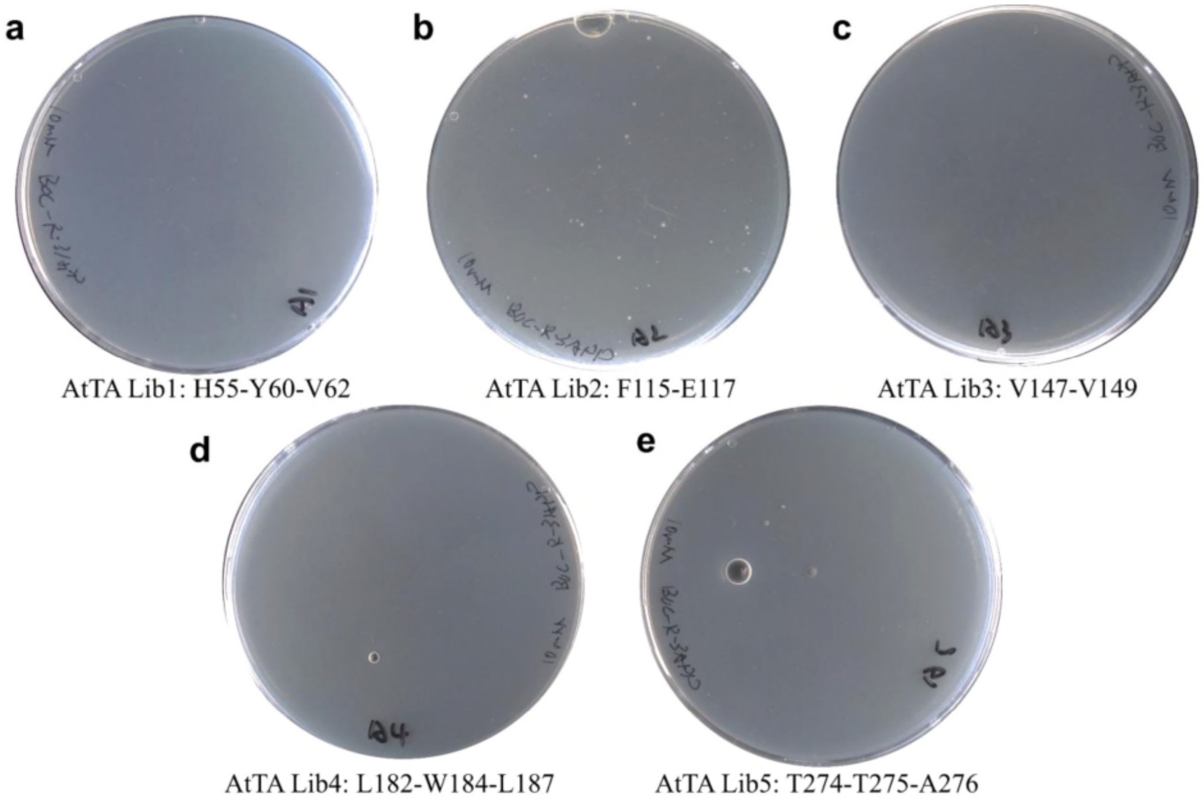

Supplementary Figure 5. Growth of *E. coli* cells containing ETm-AtTA libraries (medium expression level) on M9 agar plates with (*R*)-1 (10 mM) as the sole nitrogen source. a Growth of *E. coli* cells containing the ETm-AtTA-Lib1 (H55-Y60-V62). **b** Growth of *E. coli* cells containing the **ETm-AtTA-Lib2 (F115-E117)**. **c** Growth of *E. coli* cells containing the ETm-AtTA-Lib3 (V147-V149). **d** Growth of *E. coli* cells containing the ETm-AtTA-Lib4 (L182-W184-L187). **e** Growth of *E. coli* cells containing the ETm-AtTA-Lib5 (T274-T275-A276). The plates were incubated at 30 °C for 72 h and photos were taken then.

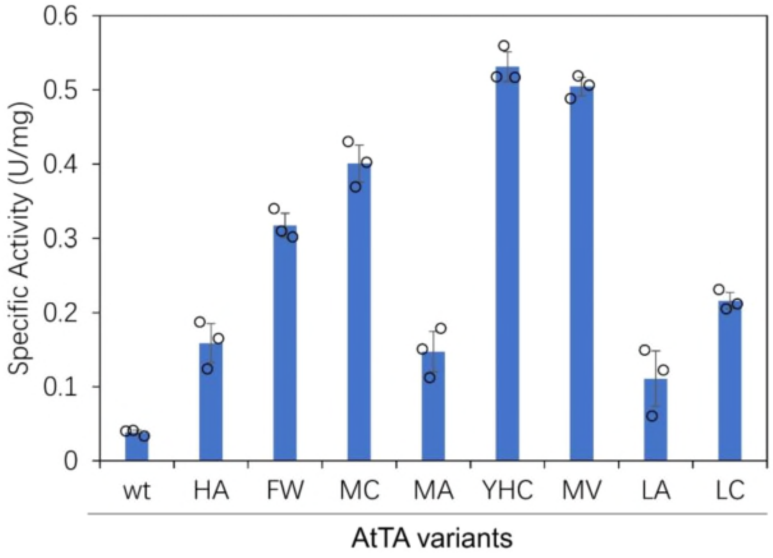

Supplementary Figure 6. Specific activities of purified AtTA variants (1st round from the F115-E117 library) towards (*R*)-1. AtTA variants and the corresponding mutations: HA, F115H/E117A; FW, E117W; **MC, F115M/E117C**; MA, F115M/E117A; **YHC, D5Y/F115H/E117C**; **MV, F115M/E117V**; LA, F115L/E117A; LA, F115L/E117C. The D5Y in the YHC variant was accidentally introduced during the mutagenesis. The specific activities of the purified AtTA variants were determined by the DAAO (D-alanine oxidase) assay. The detailed procedures of the activity assay are provided in the supplementary methods. Source data are provided as a Source Data file. Data are mean values of triplicate experiments with error bars indicating the s. d. (n = 3).

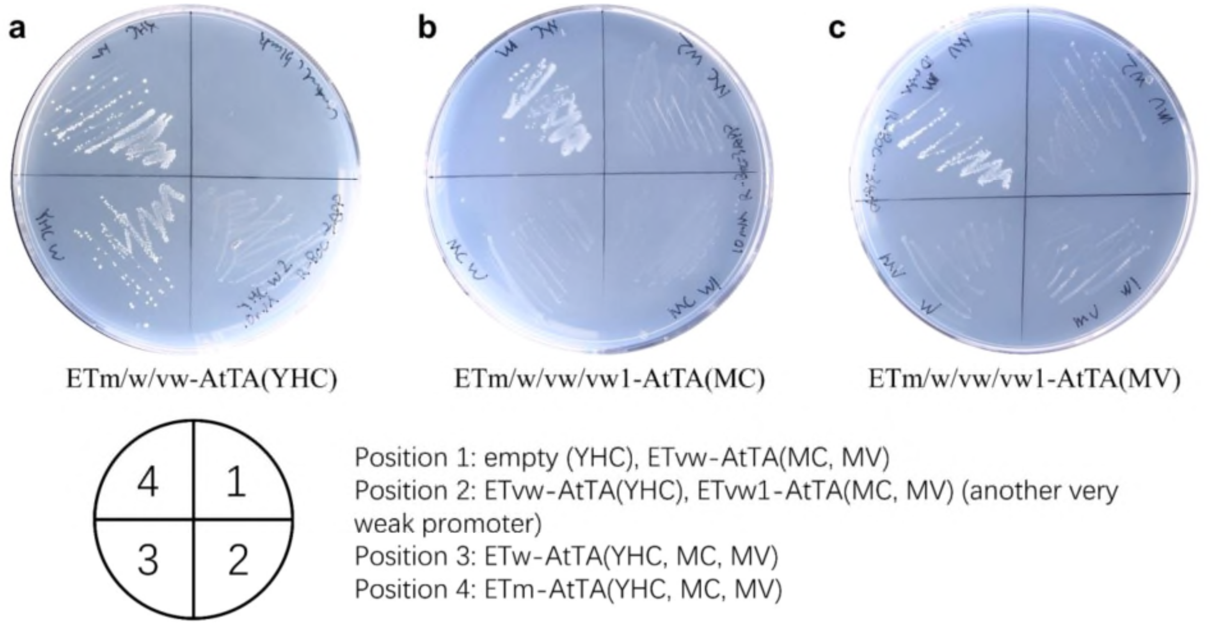

Supplementary Figure 7. Growth of *E. coli* cells containing ETm/w/vw-AtTA(MC/MV/YHC) (medium to very weak expression levels) on M9 agar plates with (*R*)-1 (10 mM) as the sole nitrogen source. a Growth of *E. coli* cells containing ETm/w/vw-AtTA(YHC) on an M9 agar plate with (*R*)-**1** (10 mM). **b** Growth of *E. coli* cells containing ETm/w/vw/vw1-AtTA(MC) on an M9 agar plate with (*R*)-**1** (10 mM). **c** Growth of *E. coli* cells containing ETm/w/vw/vw1-AtTA(MV) on an M9 agar plate with (*R*)-**1** (10 mM). For AtTA(MC) and AtTA(MV), another very weak promoter (vw1, J23117) was also used in the test. The plates were incubated at 30 °C for 72 h and photos were taken then.

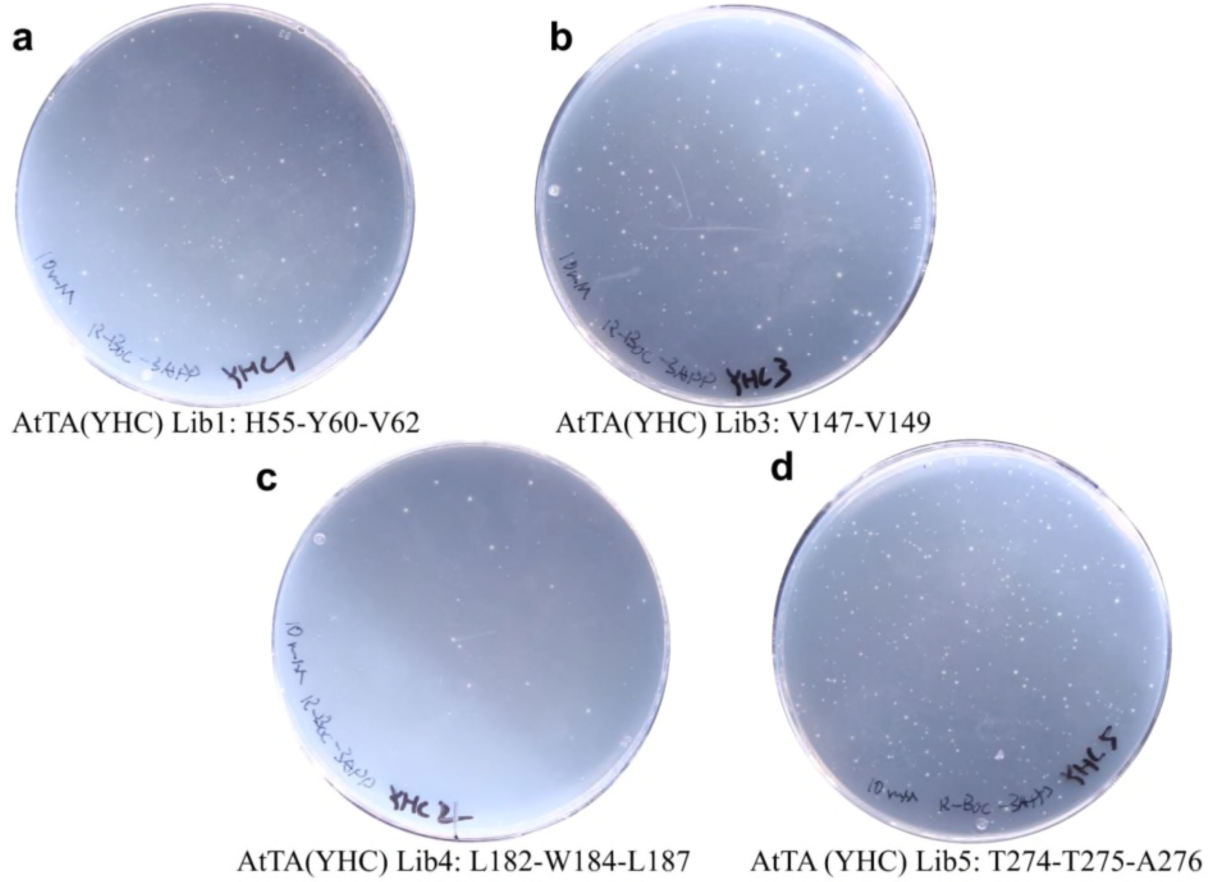

Supplementary Figure 8. Growth of *E. coli* cells containing ETvw-AtTA(YHC) libraries (very weak expression levels) on M9 agar plates with (*R*)-1 (10 mM) as the sole nitrogen source. **a** Growth of *E. coli* cells containing the ETvw-AtTA(YHC)-Lib1 (H55-Y60-V62). **b** Growth of *E. coli* cells containing the ETvw-AtTA(YHC)-Lib3 (V147-V149). **c** Growth of *E. coli* cells containing the ETvw-AtTA(YHC)-Lib4 (L182-W184-L187). **d** Growth of *E. coli* cells containing the ETvw-AtTA(YHC)-Lib5 (T274-T275-A276). The plates were incubated at 30 °C for 72 h and photos were taken then.

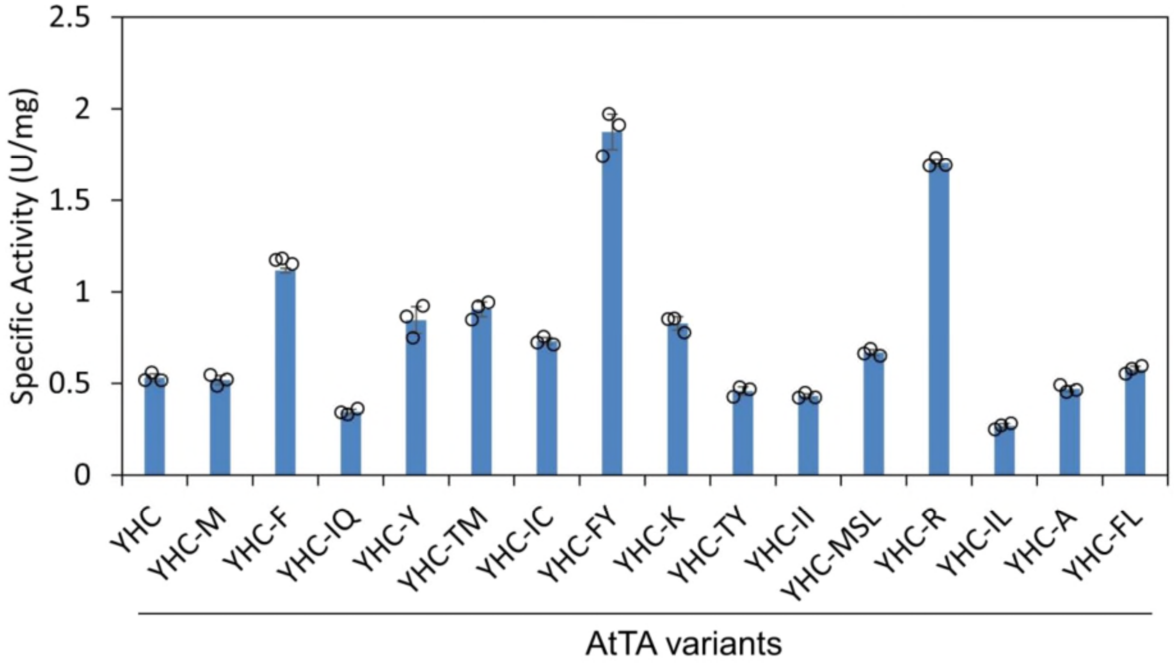

Supplementary Figure 9. Specific activities of purified AtTA variants (2nd round with YHC as the template) towards (*R*)-1. AtTA variants and the corresponding additional mutations (besides D5Y/F115H/E117C): YHC-M, L187M; YHC-F, V147F; YHC-IQ, V147I/V149Q; YHC-Y, V147Y; YHC-TM, V147T/V149M; YHC-IC, V147I/V149C; **YHC-FY, V147F/V149Y**; YHC-K, H55K; YHC-TY, V147T/V149Y; YHC-II, V147I/V149I; YHC-MSL, L73M/V147S/V149L; **YHC-R, H55R**; YHC-IL, V147I/V149L; YHC-A, T275A; YHC-FL, V147F/V149L. The specific activities of the purified AtTA variants were determined by the DAAO (D-alanine oxidase) assay. The detailed procedures of the activity assays are provided in the supplementary methods. Source data are provided as a Source Data file. Data are mean values of triplicate experiments with error bars indicating the s. d. (n = 3).

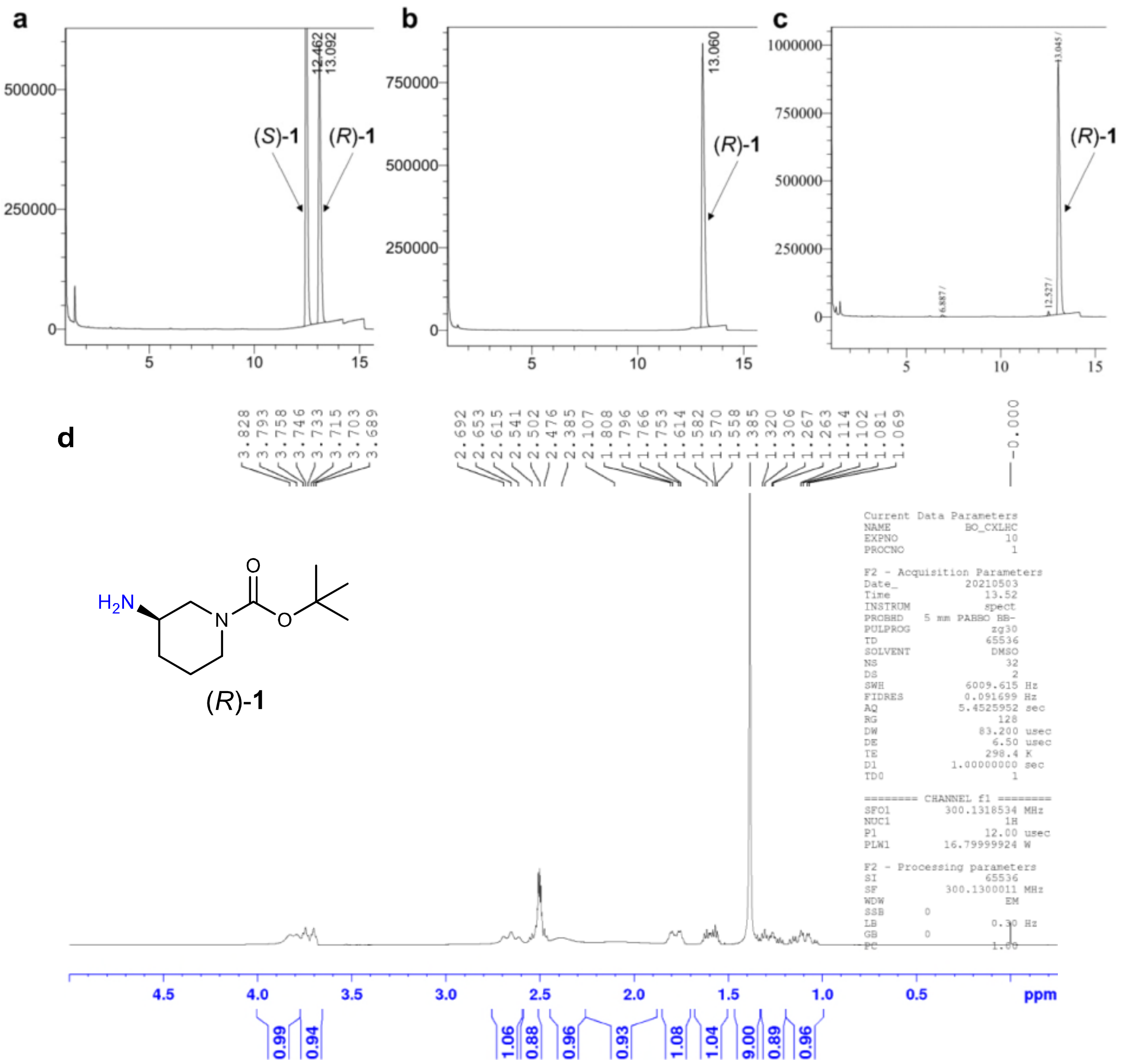

Supplementary Figure 10. Preparative scale synthesis of (*R*)-1 from 2 with purified AtTA(RHC). a Chiral GC chromatogram of the commercial standard of *rac*-**1**. **b** Chiral GC chromatogram of the commercial standard of (*R*)-**1**. **c** Chiral GC chromatogram of the synthesized (*R*)-**1**. **d** ^{1}H-NMR spectrum of the synthesized (*R*)-**1**. The detailed procedure for the preparative scale synthesis is provided in the supplementary methods.

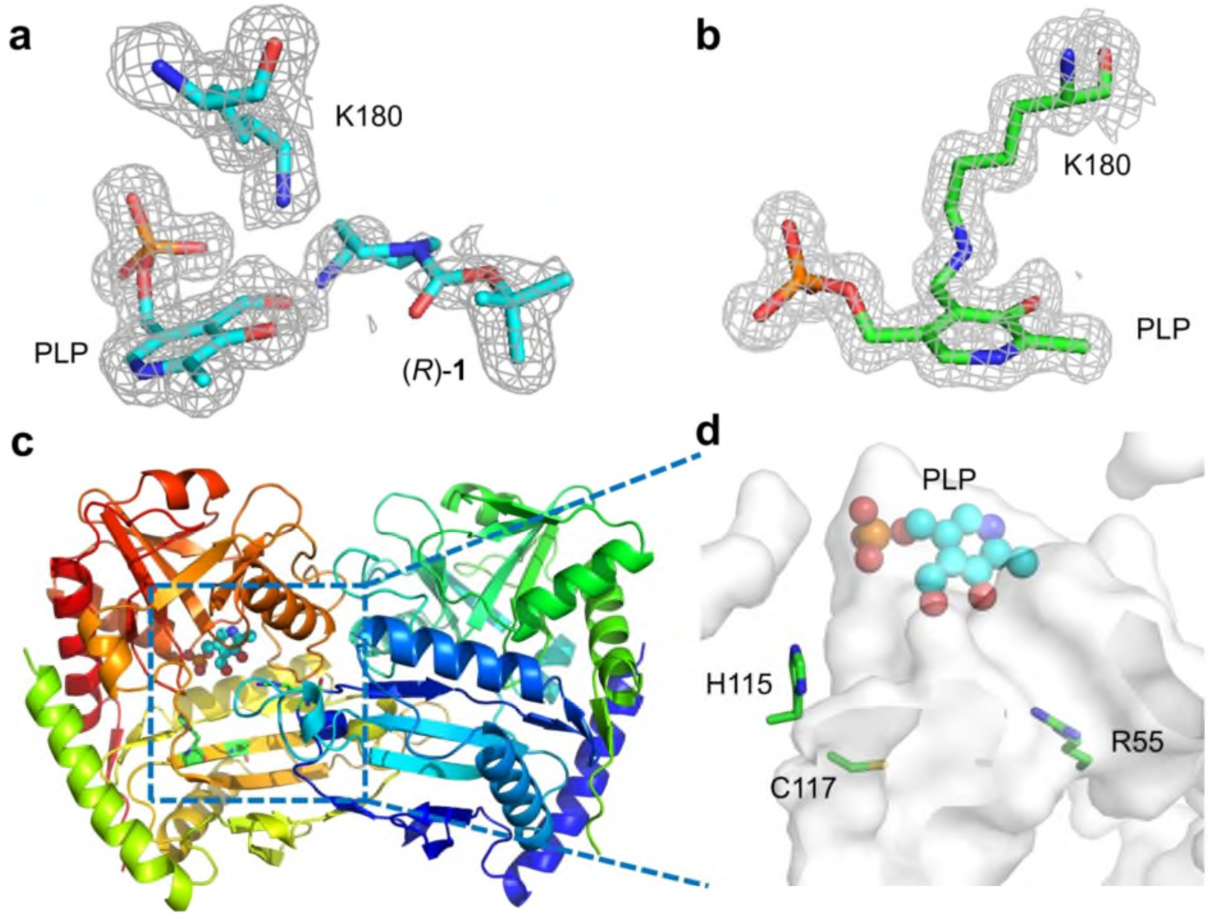

Supplementary Figure 11. Crystal structures of AtTA(RHC). a 2m|Fo|-D|Fc| map (contoured at 1.0 σ) of the AtTA(RHC) structure (PDB: 7XG5) with PLP (and a partial density as observed for (*R*)-**1**). **b** 2m|Fo|-D|Fc| map (contoured at 1.0 σ) of the AtTA(RHC) structure (PDB: 7XG6) with PLP bound to K180. **c** Overall structure of AtTA(RHC) showed a typical aminotransferase class IV fold. **d** Substrate-binding pocket of AtTA(RHC) with the mutations R55, H115, and C117. The detailed procedures of crystallization, data collection, and structure determination are provided in the supplementary methods. Data collection and refinement statistics are summarized in Supplementary Table 1.

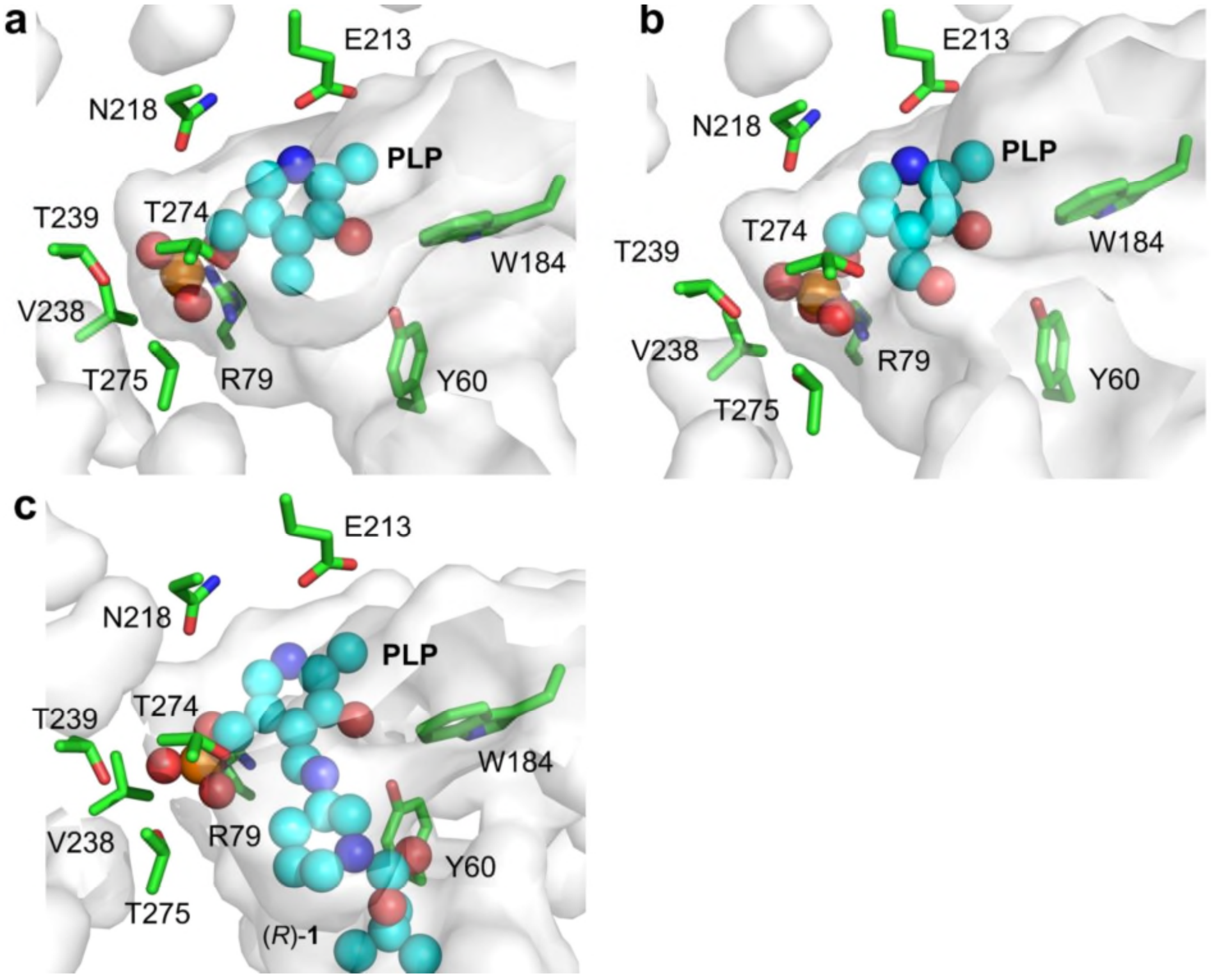

Supplementary Figure 12. PLP-binding pockets and surrounding residues. a PLP-binding pocket of AtTA(wt) (PDB: 4CE5). **b** PLP-binding pocket of AtTA(RHC) (PDB: 7XG6). **c** Binding pocket of the PLP part of the docked PLP-(*R*)-**1** complex.

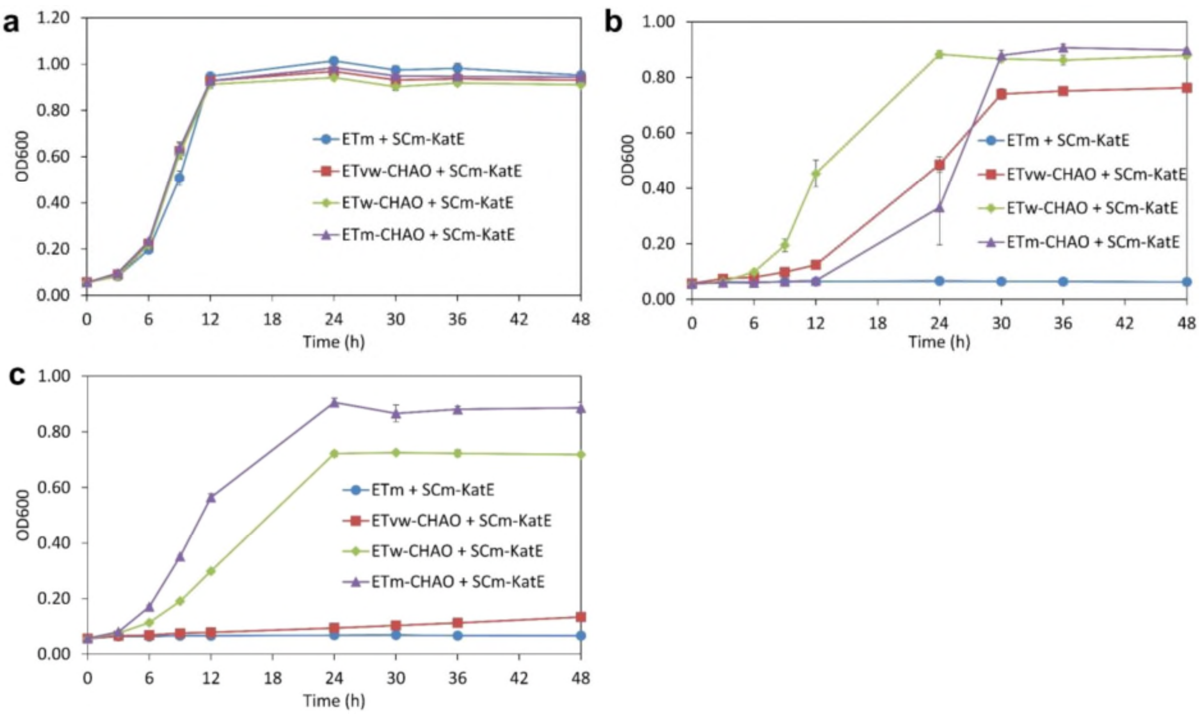

Supplementary Figure 13. Growth of *E. coli* cells co-expressing CHAO(wt) and the catalase in M9 liquid medium with different nitrogen sources. a Growth of the cells in M9 liquid medium with ammonia (10 mM) as the only nitrogen source. **b** Growth of the cells in M9 liquid medium with cyclohexylamine (10 mM, specific activity of 5.6 U mg^{-1}) as the only nitrogen source. **c** Growth of the cells in M9 liquid medium with cyclopentylamine (10 mM, specific activity of 0.33 U mg^{-1}) as the only nitrogen source. *E. coli* BL21(DE3) cells containing the ETm/w/vw-CHAO(wt) or the ETm plasmid together with the SCm-KatE plasmid (constitutive expressing of the catalase from *E. coli*) were used. ETs-CHAO(wt) with a strong promoter significantly inhibited the growth of *E. coli* even in LB medium (probably due to the burden of very strong expression), thus this was not included in this study. The cells (overnight cultured in M9 medium with ammonia) were inoculated (1%) in M9 medium (1 ml) with different nitrogen sources, and incubated in a deep 96-well plate at 30 °C and 800 rpm for 48 h. Source data are provided as a Source Data file. Data are mean values of triplicate experiments with error bars indicating the s. d. (n = 3).

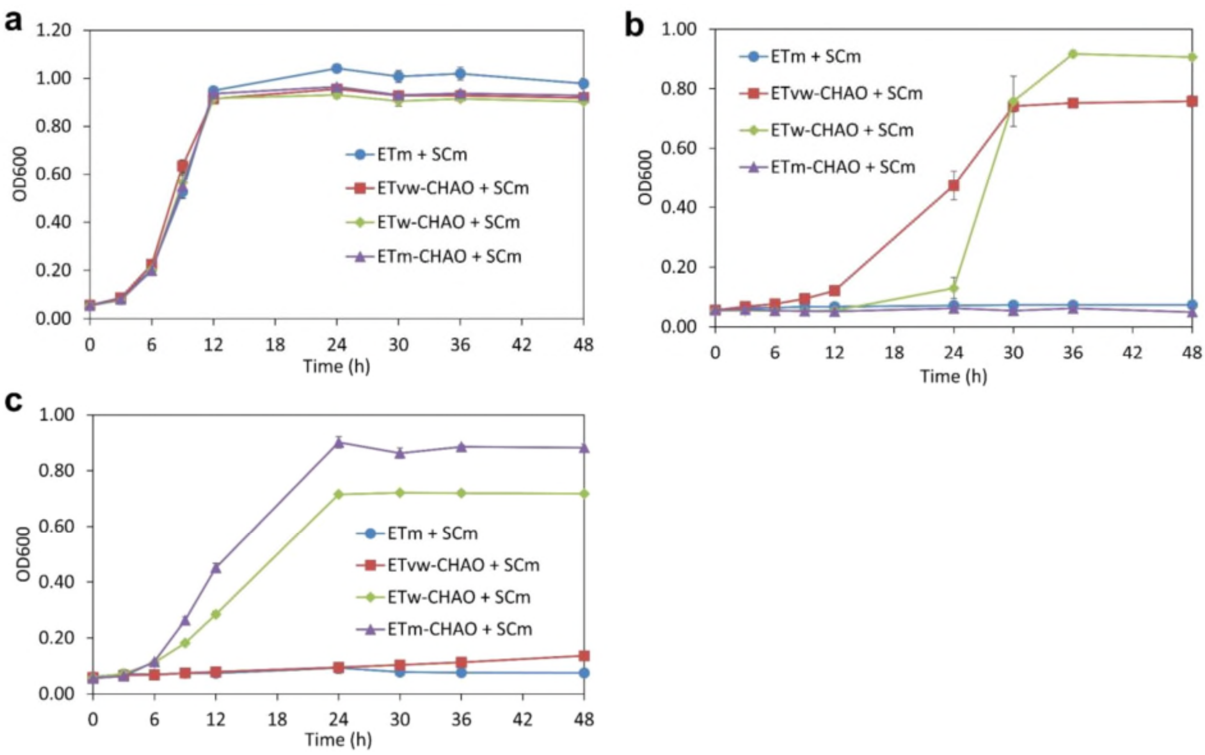

Supplementary Figure 14. Growth of *E. coli* cells expressing CHAO(wt) but without catalase in M9 liquid medium with different nitrogen sources. a Growth of the cells in M9 liquid medium with ammonia (10 mM) as the only nitrogen source. **b** Growth of the cells in M9 liquid medium with cyclohexylamine (10 mM, specific activity of 5.6 U mg^{-1}) as the only nitrogen source. **c** Growth of the cells in M9 liquid medium with cyclopentylamine (10 mM, specific activity of 0.33 U mg^{-1}) as the only nitrogen source. *E. coli* BL21(DE3) cells containing the ETm/w/vw-CHAO(wt) or the ETm plasmid together with SCm plasmid (empty, without catalase) were used. The cells (overnight cultured in M9 medium with ammonia) were inoculated (1%) in M9 medium (1 ml) with different nitrogen sources, and incubated in a deep 96-well plate at 30 °C and 800 rpm for 48 h. Source data are provided as a Source Data file. Data are mean values of triplicate experiments with error bars indicating the s. d. (n = 3).

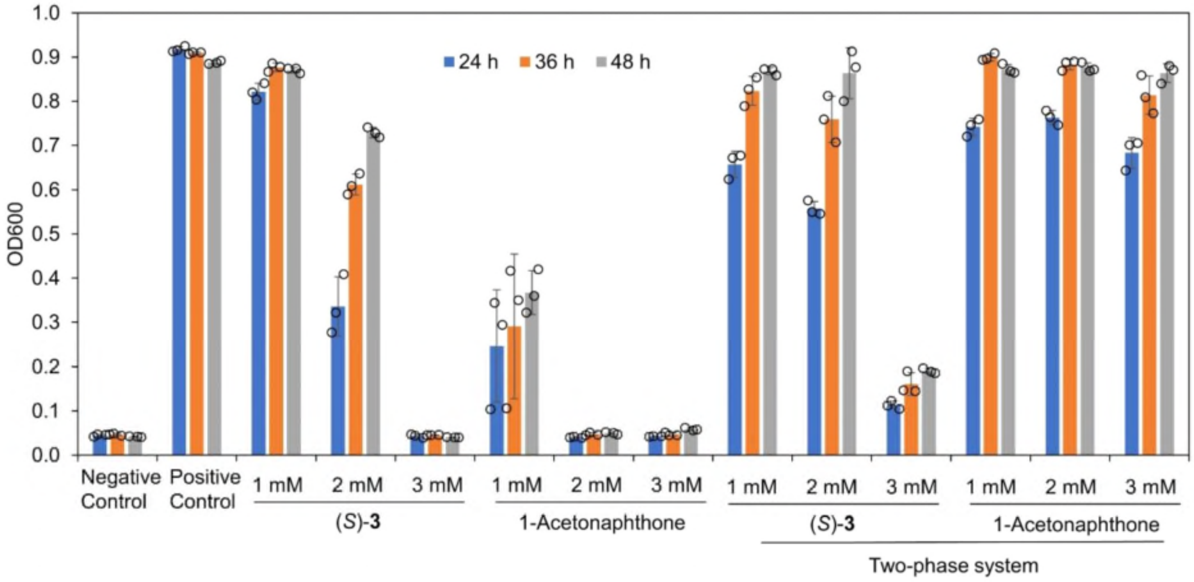

Supplementary Figure 15. Growth of *E. coli* cells in M9 liquid medium with ammonia (10 mM) and (*S*)-3 or the corresponding ketone at different concentrations. The toxicity of (*S*)-**3** or the corresponding ketone (1-acetonaphthone) was studied with *E. coli* BL21(DE3) cells containing ETm plasmid in M9 liquid medium with ammonia (10 mM). Negative control: in M9 liquid medium without nitrogen source. Positive control: in M9 liquid medium with ammonia (10 mM) without additional (*S*)-**3** or 1-acetonaphthone. Two-phase system: in M9 liquid medium with ammonia (10 mM) and methyl laurate (v/v: 4:1). The cells (overnight cultured in LB medium) were inoculated (1%) in M9 medium (2 ml) with ammonia (10 mM) and different amounts of (*S*)-**3** or 1-acetonaphthone, and incubated in cultural tubes at 30 °C and 200 rpm for 48 h. Source data are provided as a Source Data file. Data are mean values of triplicate experiments with error bars indicating the s. d. (n = 3).

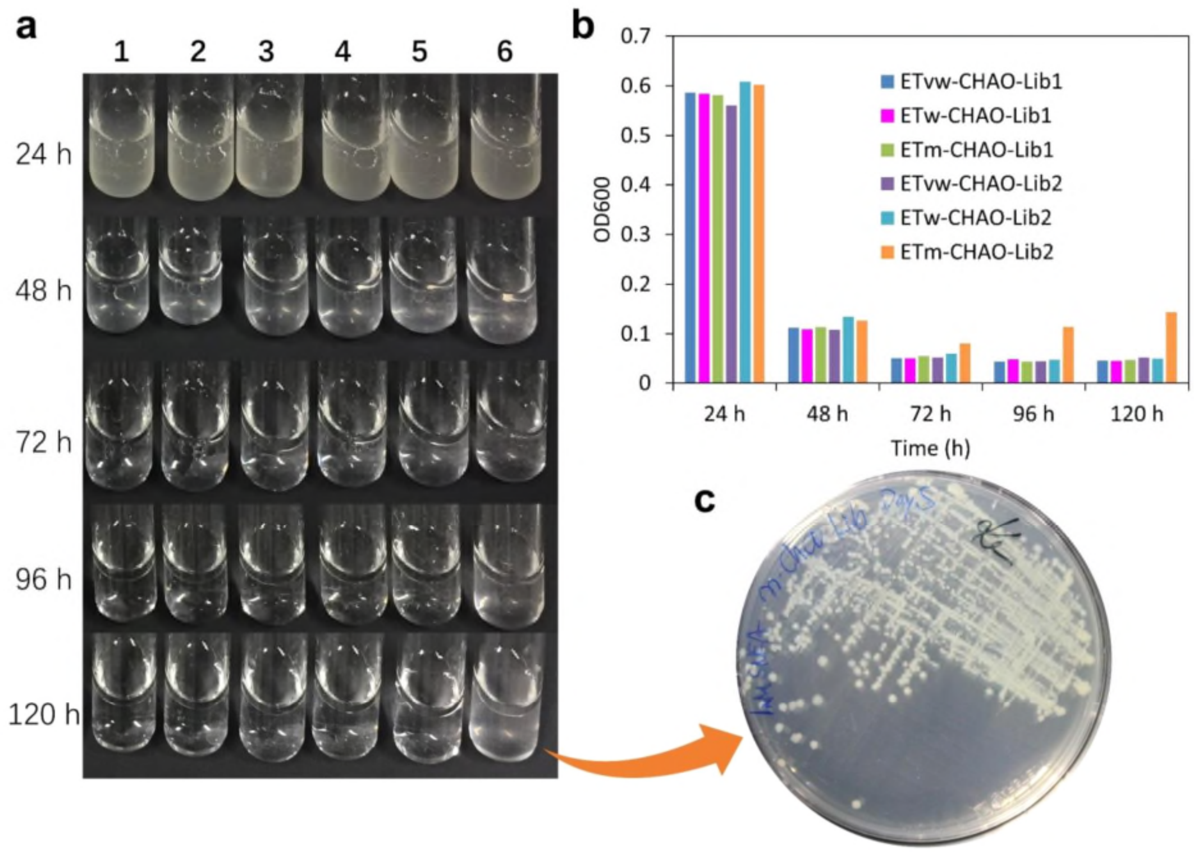

Supplementary Figure 16. Growth of *E. coli* cells co-expressing catalase and CHAO libraries in M9 liquid medium with (*S*)-3 (1 mM) and methyl laurate (v/v: 4:1). Lib1: simultaneous mutagenesis (DBS) of F88-F351-L353-F368. Lib2: simultaneous mutagenesis (DBS) of T198-L199-M226-Y321. **a** Photos of the growth of *E. coli* cells containing different CHAO libraries. Line 1: *E. coli* with ETvw-CHAO-Lib1; Line 2: *E. coli* with ETw-CHAO-Lib1; Line 3: *E. coli* with ETm-CHAO-Lib1; Line 4: *E. coli* with ETvw-CHAO-Lib2; Line 5: *E. coli* with ETw-CHAO-Lib2; Line 6: *E. coli* with ETm-CHAO-Lib2. **b** Optical density at 600 nm (OD600) of the *E. coli* cells containing different CHAO libraries. **c** Isolation of single colonies from the enriched culture of *E. coli* cells containing ETm-CHAO-Lib2 (T198-L199-M226-Y321). The detailed procedures of the growth selection are provided in the methods of the main text. Source data are provided as a Source Data file. Data in **b** are from one independent experiment.

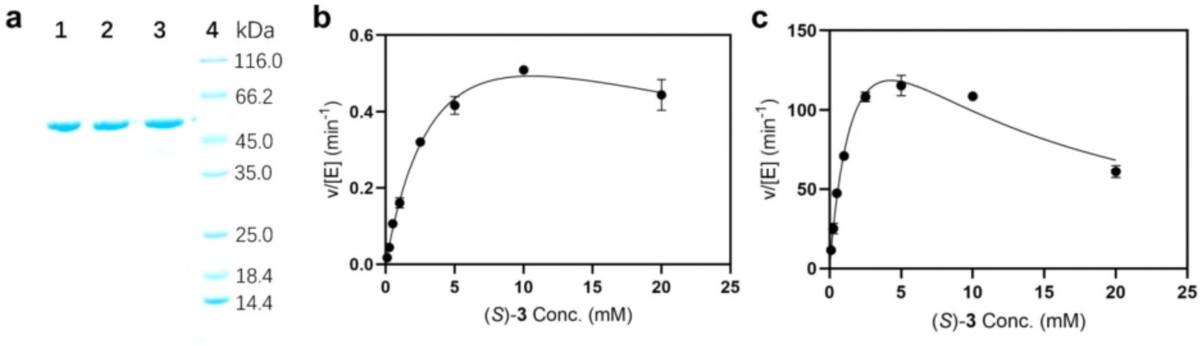

Supplementary Figure 17. Characterization of purified CHAO variants. a SDS-PAGE analysis of purified CHAO variants (with a C-terminal His-tag). Lane 1: CHAO(wt); lane 2: CHAO(MALM); lane 3: CHAO(MVLM); lane 4: protein marker. **b** Enzyme kinetics of CHAO(wt) for oxidation of (*S*)-**3**. The solid line corresponds to the fitting to the Haldane equation. **c** Enzyme kinetics of CHAO(MVLM) for oxidation of (*S*)-**3**. The solid line corresponds to the fitting to the Haldane equation. The detailed procedures for enzyme purification and kinetic data determination are provided in the supplementary methods. Source data are provided as a Source Data file. Data in **a** are from one independent experiment. Data in **b** and **c** are mean values of triplicate experiments with error bars indicating the s. d. (n = 3).

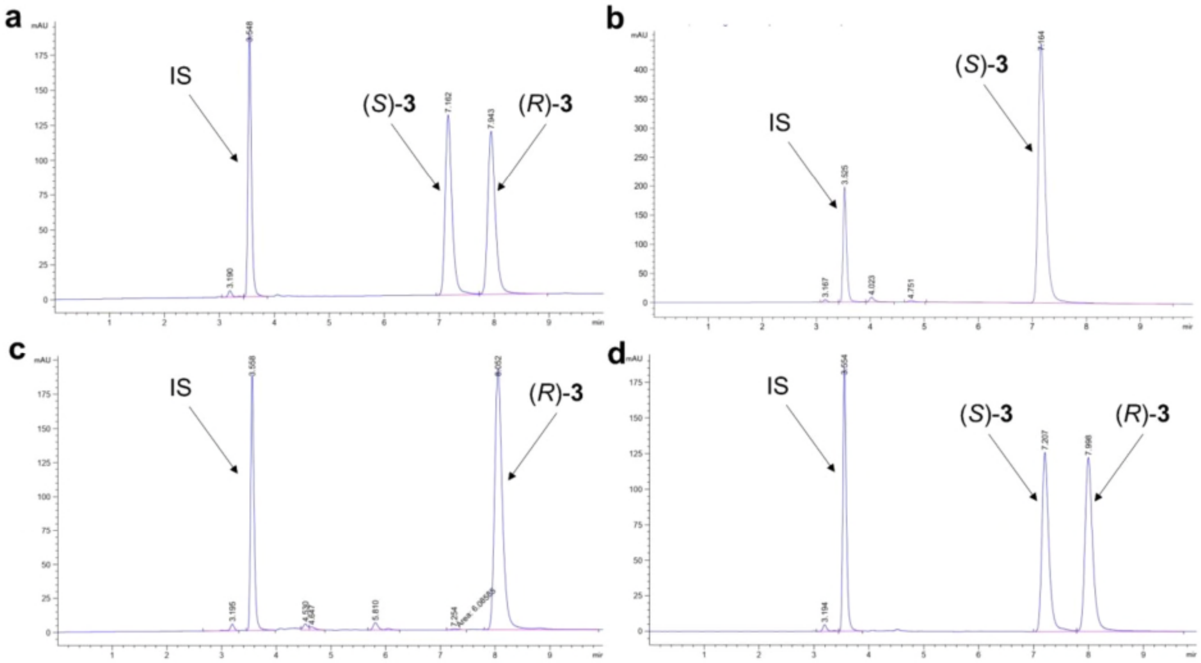

Supplementary Figure 18. Chiral HPLC chromatograms for deracemization of *rac*-3 by *E. coli* co-expressing CHAO variants and catalase and NH_3-BH_3. **a** *rac*-**3** in the reaction system without *E. coli* catalyst. **b** Commercial standard of (*S*)-**3**. **c** (*R*)-**3** produced by *E. coli* co-expressing CHAO(MVLM) and catalase and NH_3-BH_3 for 4 h. **d** *rac*-**3** left by *E. coli* co-expressing CHAO(wt) and catalase and NH_3-BH_3 for 4 h. Chiral HPLC analysis conditions: Daicel Chiralcel OD-3 column (3 µm, 4.6 mm × 250 mm), a constant flow (1.0 ml min^{-1}) of *n*-hexane (80%) and isopropanol (20%), detection at 210 nm. The detailed procedures of the reaction are provided in the methods of the main text.

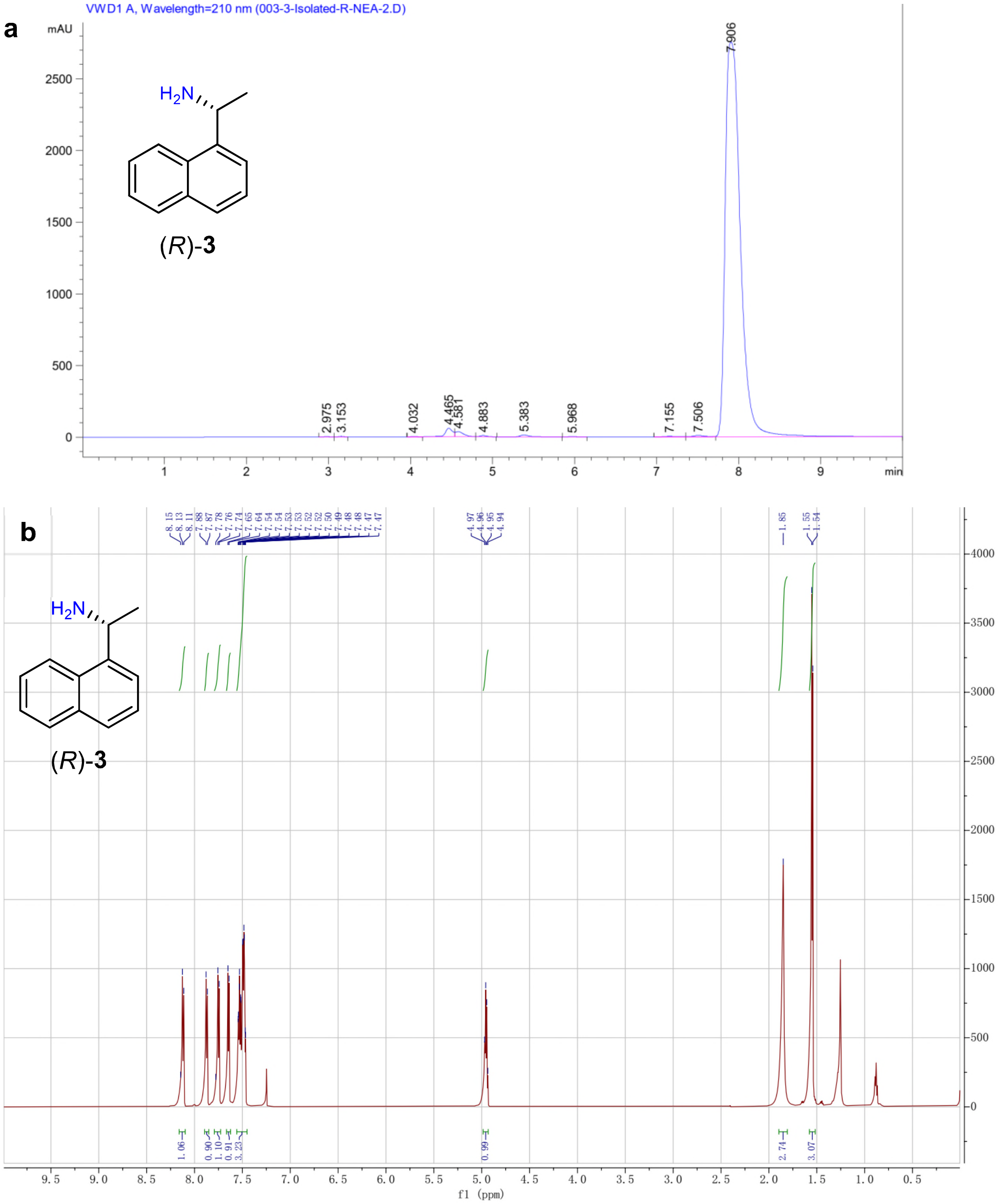

Supplementary Figure 19. Preparative scale synthesis of (*R*)-3 by deracemization of *rac*-3 by *E. coli* co-expressing CHAO(MVLM) and the catalase in the presence of NH_3-BH_3. a Chiral HPLC chromatogram of the synthesized (*R*)-**3**. **b** ^{1}H-NMR spectrum of the synthesized (*R*)-**3**. The detailed procedures for the preparative scale synthesis are provided in the supplementary methods.

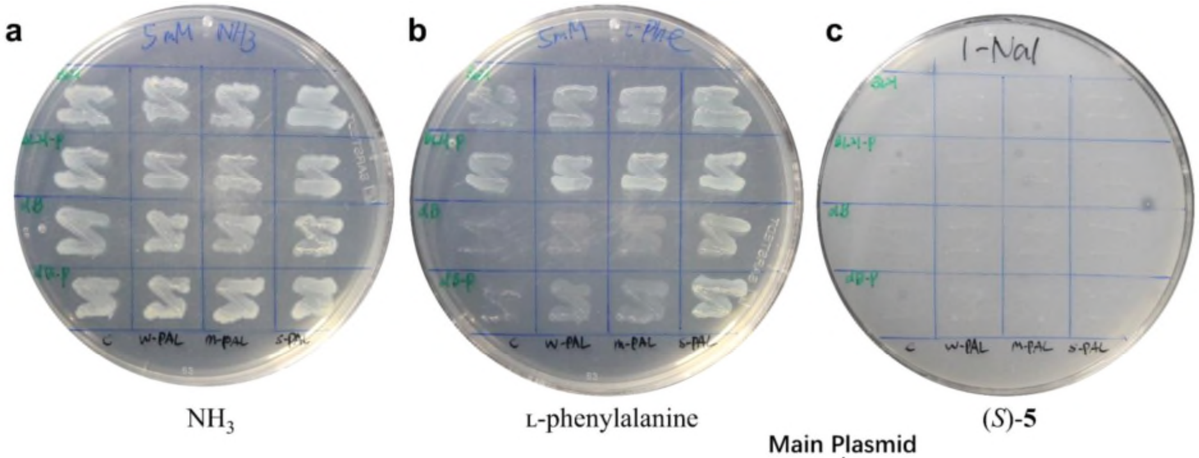

		Main Plasmid			
		ETm	ETw-PcPAL(wt)	ETm-PcPAL(wt)	ETs-PcPAL(wt)
Strain	*E.* coli BL21(DE3)	1	2	3	4
	E. coli BL21(DE3) SCm-AroP	5	6	7	8
	E. coli BL21(DE3) Δ*tyrB*	9	10	11	12
	E. coli BL21(DE3) Δ*tyrB* SCm-AroP	13	14	15	16

Supplementary Figure 20. Growth of different *E. coli* strains expressing PcPAL(wt) on M9 agar plates with different nitrogen sources. The strains differed in co-expressing the aromatic amino acid transporter (SCm-AroP) and the gene encoding the aromatic amino acid transaminase (Δ*tyrB*) was deleted. The main plasmids differed in expressing PcPAL(wt) at different levels. **a** Growth of the cells on an M9 agar plate with ammonia (5 mM) as the only nitrogen source. **b** Growth of the cells on an M9 agar plate with L-phenylalanine (5 mM) as the only nitrogen source. **c** Growth of the cells on an M9 agar plate with (*S*)-**5** (5 mM) as the only nitrogen source. Due to the limited solubility of (*S*)-**5**, the agar plate became opaque (probably because of precipitation of (*S*)-**5**) when cooling to room temperature. The *E. coli* cells containing different plasmids were streaked on the M9 agar plates with different nitrogen sources and incubated at 30 °C for 72 h and then photos were taken.

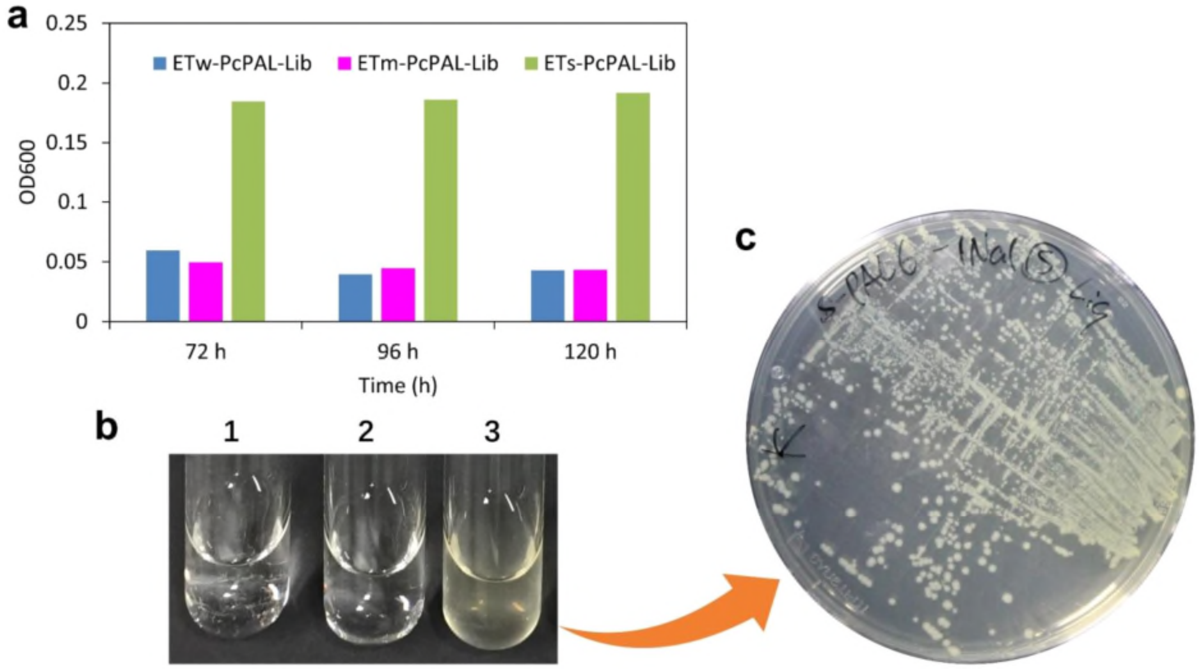

Supplementary Figure 21. Growth of *E. coli* BL21(DE3)Δ*tyrB* cells co-expressing an amino acid transporter and PcPAL libraries in M9 liquid medium with (*S*)-5 (1 mM). a Optical density at 600 nm (OD600) of the *E. coli* cells containing different PcPAL libraries. **B** Photo of the growth of *E. coli* cells containing different PcPAL libraries at 120 h. Line 1: *E. coli* with the ETw-PcPAL-Lib; Line 2: *E. coli* with the ETm-PcPAL-Lib; Line 3: *E. coli* with the ETs-PcPAL-Lib. **c** Isolation of single colonies from the enriched culture of *E. coli* cells containing the ETs-PcPAL-Lib (L134-F137-L138-L256-V259-I460). The detailed procedures of the growth selection are provided in the methods of the main text. Source data are provided as a Source Data file. Data in **a** are from one independent experiment.

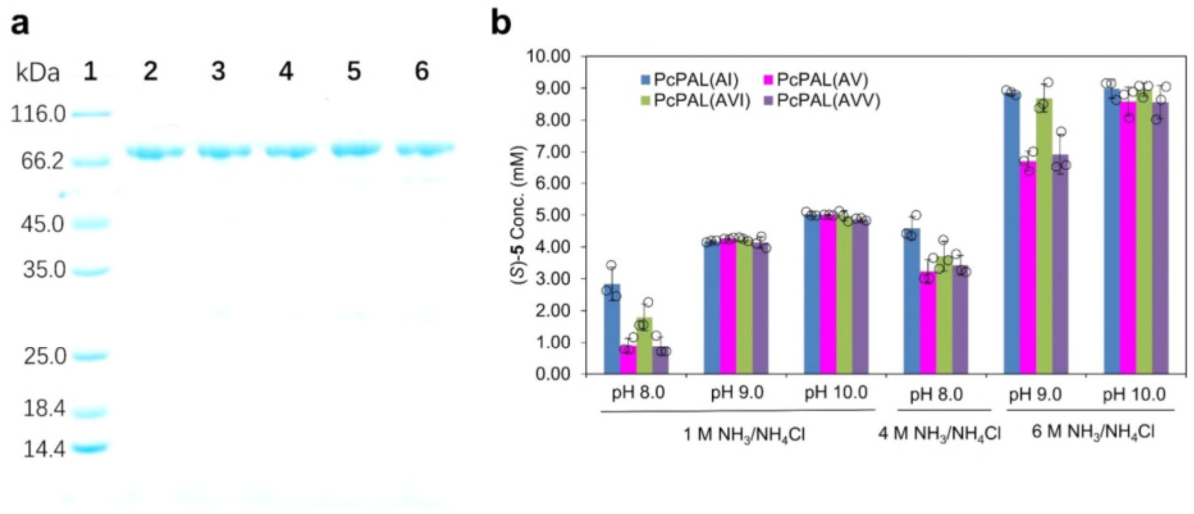

Supplementary Figure 22. Characterization of purified PcPAL variants. a SDS-PAGE analysis of purified PcPAL variants (with a C-terminal His-tag). Lane 1: protein marker; lane 2: PcPAL(wt); lane 3: PcPAL(AI); lane 4: PcPAL(AV); lane 5: PcPAL(AVI); lane 6: PcPAL(AVV). **b** Asymmetric synthesis of (*S*)-**5** from **6** by *E. coli* cells expressing PcPAL variants in different reaction buffers. Reaction conditions: **6** (10 mM), *E. coli* cells (2 g dcw l^{-1}), various buffers, 30 °C, 200 rpm, 6 h. Source data are provided as a Source Data file. Data in **a** are from one independent experiment. Data in **b** are mean values of triplicate experiments with error bars indicating the s. d. (n = 3).

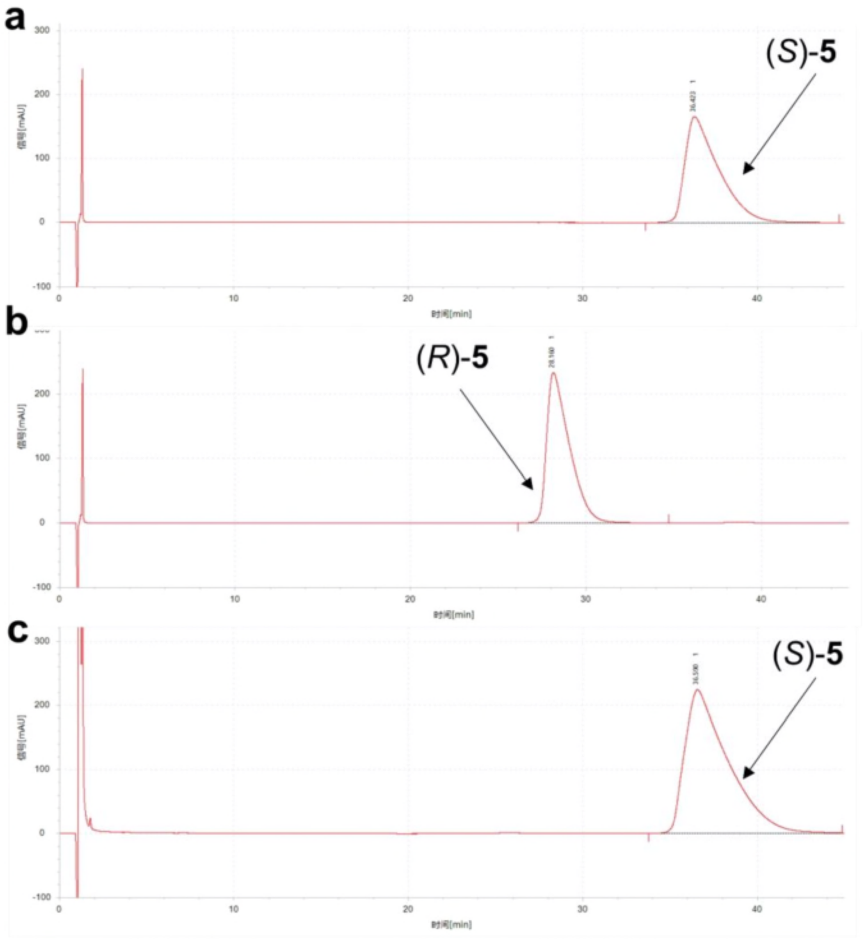

Supplementary Figure 23. Chiral HPLC chromatograms for production of (*S*)-5 from 6 by *E. coli* cells expressing PcPAL(AI). **a** Commercial standard of (*S*)-**5**. **b** Commercial standard of (*R*)-**5**. **c** (*S*)-**5** produced by *E. coli* cells expressing PcPAL(AI) for 2 h. Chiral HPLC analysis conditions: Daicel Crownpak CR(+) column (5 μm, 4.6 mm × 150 mm), a constant flow (1.0 ml min^{-1}) of H_2O (85%) containing trifluoracetic acid (0.1%) and methanol (15%), detection at 210 nm. The detailed procedures of the reaction are provided in the methods of the main text.

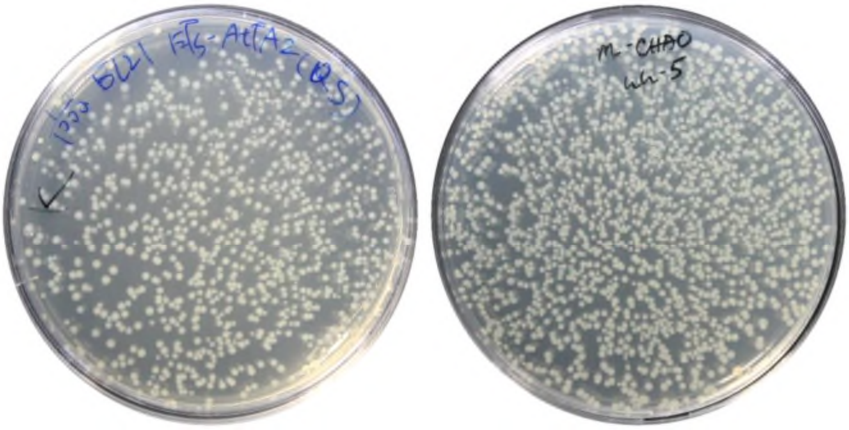

Supplementary Figure 24. Typical results of testing the electroporation efficiency by plating a small portion (1/1000) of transformed cells on LB-agar plates. The electroporation efficiency was usually 10^5-10^6 in our study.

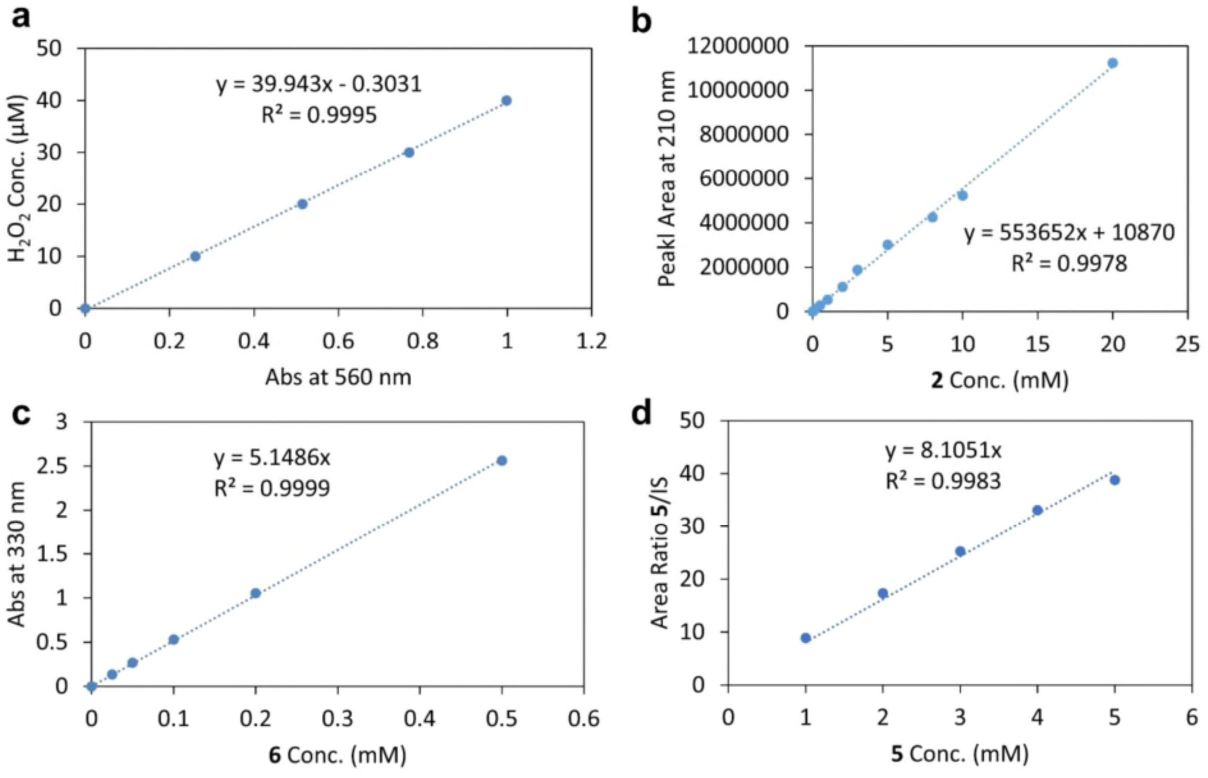

Supplementary Figure 25. Calibration curves. a Calibration curve of H_2O_2 (Ampliflu Red assay) measured using a microplate reader for the determination of the activity of AtTA and CHAO. **b** Calibration curve of **2** by HPLC. **c** Calibration curve of **6** by microplate reader. **d** Calibration curve of **5** by HPLC. Source data are provided as a Source Data file. Data in **a** are mean values of triplicate measurements. Data in **b** and **d** are from one independent measurement. Data in **c** are mean values of two measurements.

Supplementary Tables

Supplementary Table 1. Data collection and refinement statistics of AtTA(RHC) crystals.

	AtTA(RHC)-PLP[1]	AtTA(RHC)-LLP[2]
PDB code	7XG5	7XG6
**Data quality**		
space group	$C\ 2\ 2\ 2_1$	$C\ 2\ 2\ 2_1$
unit cell		
a/b/c [Å]	106.40/137.82/117.42	105.76/136.29/116.57
α /β /γ (°)	90/90/90	90/90/90
resolution [Å]	30.00 - 1.76 (1.80 - 1.76)	30.00 - 1.32 (1.34 - 1.32)
unique reflections	83896 (3657)	195796 (9406)
redundancy	13.2 (9.4)	7.4 (7.5)
completeness [%]	98.9 (93.3)	99.7 (98.9)
average $I/\sigma(I)$	11.1 (2.5)	15.9 (2.8)
CC 1/2	0.99 (0.86)	0.99 (0.84)
**Refinement**		
R_{work}[a] (95 % of data)	0.159 (0.237)	0.154 (0.208)
R_{free}[a] (5 % of data)	0.181 (0.270)	0.163 (0.231)
r.m.s.d. bonds [Å]	0.019	0.016
r.m.s.d. angles [º]	1.73	1.62
dihedral angles		
most favored [%]	98.12	98.26
allowed [%]	1.88	1.74
disallowed [%]	0	0
**Average B-factor/ Number of non-hydrogen atoms**		
Protein	21.6/5010	14.0/5030
Ion/ligands	16.4/32	11.8/48
solvent	34.4/899	28.0/1031

[1] AtTA(RHC) with PLP in the cavity (not covalent bound Lys180) was prepared under soaking conditions.

[2] AtTA(RHC) with covalently bound LLP (PLP-Lys180) was prepared under whole structure conditions.

Values in parentheses are for the highest resolution shell.

Supplementary Table 2. List of key primers used in this study.

Name	Sequence
pRSF1623-R	TCAAATATGTATCCGCTCATGAGAC
pRSF2637-F	TCGTGCCAGCTGCATTAATGAAT
pET2022-F	GTCTCATGAGCGGATACATATTTGA
pET3983-R	ATTCATTAATGCAGCTGGCACGA
J23100S-F	cctaggtacagtgctagcTAATTTTGTTTAACTTTAATAAGGAG
J23100S-R	actgagctagccgtcaaATTCATTAATGCAGCTGG
J23106M-F	cctaggtatagtgctagcTAATTTTGTTTAACTTTAATAAGGAG
J23106M-R	actgagctagccgtaaaATTCATTAATGCAGCTGGCA
J23116W-F	ggactatgctagcTAATTTTGTTTAACTTTAATAAGGAG
J23116W-R	ctaggactgagctagctgtcATTCATTAATGCAGCTGGC
J23109VW-F	cctagggactgtgctagcTAATTTTGTTTAACTTTAATAAG
J23109VW-R	actgagctagctgtaaaATTCATTAATGCAGCTGGC
AtTAopt-F	taataaggagatatattATGGCATCAATGGATAAGGTTTTTGCCGGTTATG
AtTA-R	gtttctttaccagactcgagTCAATGATGATGATGATGATGGG
Duet-TT1stATG-F	GTTTAACTTTAATAAGGAGATATATTATG
Duet-TT1stATG-R	CATAATATATCTCCTTATTAAAGTTAAAC
Duet-1stATG-F	GTTTAACTTTAATAAGGAGATATACCATG
Duet-1stATG-R	CATGGTATATCTCCTTATTAAAGTTAAAC
Duet-XhoI-F	CTCGAGTCTGGTAAAGAAACCG
Duet-XhoI-R	CGGTTTCTTTACCAGACTCGAG
AtTA-6062X-F	tgaccnnkgatnnkCCGAGCGTTTGGGATG
AtTA-55X-R	gatcgctmnnCATAAAACCCTGATCCAGCAG
AtTA-117X-F	nnkCTGATTGTTACCCGTGGTC
AtTA-115X-R	cacmnnTGCATCACGAATACCGC
AtTA-149X-F	tggnnkATGGAACCGGATATGCAG
AtTA-147X-R	mnnATACGGCTGCACAAACATATAC
AtTA-1847X-F	nnkggtgatnnkGTTCGTGGTATGTTTGAAGC
AtTA-182X-R	ctgmnnATTTTTAACGGTCGGATCAATTG
AtTA-2756X-F	nnknnkGGCGGTATTATGCCGATTAC
AtTA-274X-R	mnnACACATAAAAATTTCATCACAGCG
CHAO-F	taataaggagatataccATGACTCACCTGAATACCTATGAG
CHAO-R	gtttctttaccagactcgagTTAGTGGTGGTGATGGTGATG
ET-dBsaI-F	ttGGTCTCagactCCGCAAGTGGCACTTTTCG
ET-dBsaI-R	ttGGTCTCaagtcCCGGTCGTCAGCTTGTC
CHAO-88-R	ttGGTCTCaCATACCACCGTAATCAATTGGAC
CHAO-88-F	ttGGTCTCaTATGdbsATCGGTGAGACGCATACCC
CHAO-3513-R	ttGGTCTCaCACTCCCACATCCTCGGTATC
CHAO-3513-F	ttGGTCTCaAGTGdbsTTAdbsGACGGAACTAAGCCCACCG
CHAO-368-R	ttGGTCTCaGCCGATGAGAGTGGCGAG
CHAO-368-F	ttGGTCTCaCGGCdbsATTGGCGGCAGCAACTAC
CHAO-1989-R	ttGGTCTCaATTGACGATTACGGTGTGAAG
CHAO-1989-F	ttGGTCTCaCAATdbsdbsTTAGGCGCTGATCCTTACG
CHAO-226-R	ttGGTCTCaGAGTGACTGGATGCCCTCAC

CHAO-226-F	ttGGTCTCaACTCdbsGGGACGCGCGACGGTG
CHAO-321-R	ttGGTCTCaACGACCCATCGGGGCGCG
CHAO-321-F	ttGGTCTCaTCGTdbsTATAAAGTCCAAGCACGCTACC
PcPAL-F	taataaggagatataccATGGAAAACGGTAACGGCGC
PcPAL-His-R	ttagtggtggtgatggtgatgGCAGATCGGCAGCGGAG
Duet-His-XhoI-F	catcaccatcaccaccactaaCTCGAGTCTGGTAAAGAAAC
PcPAL-704S-R	ccattccaggattccagggATTCCAGCAGCGGGTCGA
PcPAL-716S-F	tgctccgctgccgatctcCCATCACCATCACCACCAC
PcPAL-1378-F	GCdycdyaAACGCTGGTATCTTCGGTAAC
PcPAL-134-R	GGATtrhTTCTTTCTGCAGTGCACCC
PcPAL-259-F	CTGrycAACGGTACCGCTGTTGG
PcPAL-256-R	TGCtrhGCCCTCTTTCGGTTGCAG
PcPAL-460-F	CTATGGCTTCCTACTGTTCTGAAC
PcPAL-460-R	CgryTTCTGCACCTTTGAAACCATAGTC
EcKatE-F	taataaggagatataccATGTCGCAACATAACGAAAAGAAC
EcKatE-R	gtttctttaccagactcgagTTAGGCAGGAATTTTGTCAATCTTAG
EcAroP-F	taataaggagatataccATGATGGAAGGTCAACAGCAC
EcAroP-R	tctttaccagactcgagTTAATGCGCTTTTACGGCTTTG
tyrB-Up-F	gagtcgacctgcagaagcttGTAAGCGAACGTGATACCCGTC
tyrB-Up-R	ctttcctgcaGCGATGGTTCTCCAGGTTTACG
tyrB-Down-F	gaaccatcgcTGCAGGAAAGCAGGCTGGAG
tyrB-Down-R	gagctgcacatgaactcgagCACGCTTTGCTGTTTTGCCGAG
pTarget-F	CTCGAGTTCATGTGCAGCTC
pTarget-R	AAGCTTCTGCAGGTCGACTC
dTyrB-gRNA-F	ggcggctccgGTTTTAGAGCTAGAAATAGCAAGTT
dTyrB-gRNA-R	aagggtttgaACTAGTATTATACCTAGGACTGAGC

Supplementary Methods

Chemicals, Materials, and Software

All chemicals were purchased from commercial suppliers and used without further purification. The key chemicals are listed below.

Chemicals from BLD pharma purchased from ChemPur: (*R*)-1-*Boc*-3-aminopiperidine (*R*)-**1** (BD0329, 97%, CAS 188111-79-7), (*S*)-1-*Boc*-3-aminopiperidine (*S*)-**1** (BD0332, 97%, CAS 625471-18-3), (*R*)-1-(1-naphthyl)ethylamine (*R*)-**3** (BD20592, 98%, CAS 3886-70-2), (*S*)-1-(1-naphthyl)ethylamine (*S*)-**3** (BD17383, 98%, CAS 10420-89-0), 3-(1-naphthyl)-L-alanine (*S*)-**5** (BD14666, 97%, CAS 55516-54-6), 3-(1-naphthyl)-D-alanine (*R*)-**5** (BD14665, 97%, CAS 78306-92-0), D-alanine (BD33271, 97%, CAS 338-69-2), borane-ammonia complex (BD118552, 98%, CAS 13774-81-7).

Chemicals purchased from Enamine: 1-*Boc*-3-piperidinone **2** (EN300-55665, 95%, CAS 98977-36-7), (2*E*)-3-(naphthalen-1-yl)prop-2-enoic acid **6** (EN300-833017, 95%, CAS 2006-14-6).

Chemicals from Acros Organics: 1-(1-naphthyl)ethylamine *rac*-**3** (450570050, 98%, CAS 42882-31-5), cyclohexylamine (111282500, 98%, CAS 108-91-8), cyclopentylamine (111590250, 98%, CAS 1003-03-8).

Chemicals from ABCR: (*R*)-1-phenylethylamine (AB118494, 98%, CAS 3886-69-9).

Chemicals from Sigma-Aldrich: 1-acetonaphthone (67990, 97%, CAS 941-98-0), methyl laurate (61689, 99%, CAS 111-82-0), L-phenylalanine (P2126, 99%, CAS 63-91-2), Ampliflu™ Red (90101, 98%, CAS 119171-73-2), horseradish peroxidase (P8125, HRP).

T4 DNA ligase (EL0011) and DNA primers were purchased from Thermo Fisher.

Q5 high fidelity DNA polymerase (M0491), KLD Enzyme mix (M0554), and BsaI-HF v2 (R3733), were purchased from New England Biolabs.

The plasmid miniprep kit (740588) and the gel extraction kit (740609) were bought from Macherey-Nagel.

DNA sequencings were performed by Eurofins.

Microsoft Excel was used to analyze conversion and activity and draw charts. GraphPad Prism was used to analyze enzyme kinetic data. MestReNova was used for analysis of NMR data. ChemDraw was used to draw the schemes. Microsoft Power Point was used to prepare and combine figures. Other software was specified in the following sections.

Culture media

Medium for routine molecular biology works of *E. coli*: LB (Lysogeny broth) medium containing tryptone (10 g l^{-1}), NaCl (5 g l^{-1}) and yeast extract (5 g l^{-1}).

Medium for expression of enzymes in *E. coli* for purification of enzymes and whole-cell reactions: TB (Terrific broth) medium containing tryptone (12 g l^{-1}), yeast extract (24 g l^{-1}), glycerol (4 ml l^{-1}), K_2HPO_4 (12.5 g l^{-1}), and KH_2PO_4 (2.3 g l^{-1}).

Medium for growth selection of active amine enzymes in *E. coli* cells: modified M9 medium containing Na_2HPO_4 (6 g l^{-1}), KH_2PO_4 (3 g l^{-1}), NaCl (0.5 g l^{-1}), $MgSO_4$ (1 mM), $CaCl_2$ (0.1 mM), biotin (1 mg l^{-1}), thiamine (1 mg l^{-1}), trace metal solution (1 ml l^{-1} of 1000× stock solution, see below), glucose (4 g l^{-1}, carbon source), and the specific nitrogen source (1-10 mM).

Trace metal solution (1000× stock solution) containing $FeCl_3$ (0.5 g), $ZnSO_4{\cdot}7H_2O$ (0.15 g), $MnCl_2{\cdot}4H_2O$ (50 mg), $CuCl_2{\cdot}2H_2O$ (20 mg), $CoCl_2{\cdot}6H_2O$ (30 mg), $Na_2MoO_4{\cdot}2H_2O$ (30 mg), and H_3BO_3 (30 mg) in HCl solution (0.1 M, 50 ml).

Additional agar (15 g l^{-1}) was included for the preparation of solid medium for agar plates.

Appropriate amount of antibiotics was used: kanamycin (50 mg l^{-1}) and ampicillin (25 mg l^{-1}) for cultures in LB or TB medium. The concentration was reduced to half during the selection in M9 medium.

DNA Sequences

The AtTA gene was reported in our previous report.[1] It was engineered to add a C-terminal His-tag.

DNA sequence:

>AtTA

ATGGCATCAATGGATAAGGTTTTTGCCGGTTATGCAGCACGTCAGGCAATTCTGGAAAGCACCGAAACCACCAATC
CGTTTGCAAAAGGTATTGCATGGGTTGAAGGTGAACTGGTTCCGCTGGCAGAAGCACGTATTCCGCTGCTGGATC
AGGGTTTTATGCATAGCGATCTGACCTATGATGTTCCGAGCGTTTGGGATGGTCGTTTTTTTCGTCTGGATGATCAT
ATTACCCGTCTGGAAGCCAGCTGTACCAAACTGCGTCTGCGTCTGCCGCTGCCTCGTGATCAGGTTAAACAAATTC
TGGTTGAAATGGTTGCCAAAAGCGGTATTCGTGATGCATTTGTGGAACTGATTGTTACCCGTGGTCTGAAAGGTGT
TCGTGGCACCCGTCCGGAAGATATCGTGAATAATCTGTATATGTTTGTGCAGCCGTATGTTTGGGTTATGGAACCG

GATATGCAGCGTGTTGGTGGTAGCGCAGTTGTTGCACGTACCGTTCGTCGTGTTCCGCCTGGTGCAATTGATCCGA
CCGTTAAAAATCTGCAGTGGGGTGATCTGGTTCGTGGTATGTTTGAAGCAGCAGATCGTGGTGCAACCTATCCGTT
TCTGACCGATGGTGATGCACATCTGACCGAAGGTAGCGGTTTTAACATTGTGCTGGTGAAAGATGGTGTTCTGTAT
ACACCGGATCGTGGTGTTCTGCAGGGTGTTACACGTAAAAGCGTGATTAATGCAGCAGAAGCCTTTGGTATTGAA
GTGCGTGTTGAATTTGTTCCGGTTGAACTGGCATATCGCTGTGATGAAATTTTTATGTGTACCACCGCAGGCGGTA
TTATGCCGATTACCACCCTGGATGGTATGCCGGTTAATGGTGGTCAGATTGGTCCGATTACCAAAAAAATTTGGGA
TGGCTATTGGGCAATGCATTATGATGCAGCCTATAGCTTTGAAATTGATTATAATGAACGCAATTCAGGATCCCAT
CATCATCATCATCATTGA

The CHAO gene originating from *Brevibacterium oxydans* IH-35A[2] was codon-optimized for *E. coli*, a C-terminal His-tag was added, and it was synthesized by Twist Bioscience. Optimized DNA sequence:

>CHAO

ATGACTCACCTGAATACCTATGAGAGTGTGACCCCGGACCCGGATGTAGATGTAATTATTATTGGCGCAGGCATCT
CCGGTAGCGCAGCGGCTAAGGCGCTGCACGACCAGGGAGCCAGCGTCCTGGTTGTTGAGGCGAATGACCGTATT
GGCGGACGTACGTGGACCGAGCAAGAGGGAGCACCCGGTGGTCCAATTGATTACGGTGGTATGTTCATCGGTGA
GACGCATACCCATCTGATTGAGCTTGGCACGTCTCTTGGTCTGGAGATGACGCCATCGGGGAAGCCAGGAGATGA
TACTTATATTGTTGCAGGGAATGTTCTGCGTGCACCAGACGACCAACTCGACCCTAATCTCCCTTTTGTGCCGGAGT
TTCTTAGCTCGTTAAAGGCCCTGGACGAGCTTGCGGATAGTGTAGGCTGGGACCAACCGTGGGCCAGTCCAAACG
CAGCCGCGTTAGACAGTAAGACCGTGGCCACCTGGCTCGCGGAAACCATCGAAAGCGAAGAGGTCCGTCGCCTTC
ACACCGTAATCGTCAATACCCTCTTAGGCGCTGATCCTTACGAGGTATCGCTCCTGTATTGGGCTTATTATGTGAGC
GAGTGTGAGGGCATCCAGTCACTCATGGGGACGCGCGACGGTGCACAATGGGCATGGTGGTTCGGTGGTGCCGC
GCAAGTGAGTTGGCGTATTGCTGACGCAATTGGGCGCGATAAGTTCTTACTGGAGTGGCCGGTAGACCGCATCGA
GCACGACGAGAGCGGGGTGACATTGTTCTCAGGGCAACGCTCACTGCGCGCTCGTCACATTGTGATTGCAATGAG
CCCTCTTGCGGCGAACCAGATCCGCTTCGAGCCGGCCCTGCCGACTTCTCGTGCCCAGCTGCAAGCACGCGCCCCG
ATGGGTCGTTATTATAAAGTCCAAGCACGCTACCCATCATCCTTTTGGGTAGAGCAAGGTTATTCGGGCGCGTTGC
TGGATACCGAGGATGTGGGAGTGTTCTTACTGGACGGAACTAAGCCCACCGACACCCTCGCCACTCTCATCGGCTT
TATTGGCGGCAGCAACTACGATCGTTGGGCGGCTCATACACCTCAAGAGCGTGAGCGCGCATTTCTTGACTTGTTA
GTGAAGGCATTCGGGCCACAAGCGGCCGACCCTTCATACTTTCACGAAACCGACTGGACTCAGCAAGAGTGGGCC
AAGGGCGGTCCAGTCACTTATATGCCGCCTGGAGTTTTGGCGAATTTCGGTGCCGCGCTCCGCGATCCAGTCGGC
AAGGTCCATTTTGCGGGTACTGAGGCATCATTCCAATGGTCCGGCTACATGGAAGGTGGCGTCCGCGCCGGCCAA

AAGGCCGCCGCGGCGATTGCGGAAGAACTCGAACGCACGGCGAACAAGGGAGCGTTAGTTCATCACCATCACCA
CCACTAA

The PcPAL gene was described in our previous report.[3] A C-terminal His-tag was added. DNA sequence:

>PcPAL

ATGGAAAACGGTAACGGCGCTACCACTAACGGTCACGTGAACGGCAACGGTATGGACTTCTGCATGAAAACCGAA
GATCCTCTGTACTGGGGCATCGCTGCGGAGGCTATGACTGGTTCCCACCTGGACGAAGTTAAAAAGATGGTTGCT
GAATATCGTAAACCGGTTGTTAAACTGGGTGGCGAAACTCTGACCATCTCCCAAGTTGCTGCAATCTCTGCTCGTG
ACGGTTCCGGTGTTACTGTTGAACTGTCCGAAGCTGCGCGTGCTGGTGTTAAAGCGTCCTCTGACTGGGTTATGGA
CTCCATGAACAAAGGTACCGACTCCTATGGCGTTACCACTGGTTTCGGCGCTACCTCCCATCGTCGTACCAAACAG
GGGGGTGCACTGCAGAAAGAACTGATCCGCTTCCTGAACGCTGGTATCTTCGGTAACGGTTCTGACAATACGCTG
CCGCATTCCGCTACCCGTGCTGCTATGCTGGTTCGTATCAACACCCTGCTGCAAGGTTACTCTGGTATCCGTTTCGA
AATCCTGGAGGCTATCACGAAATTCCTGAACCAGAACATCACCCCGTGCCTGCCGCTGCGTGGTACCATCACTGCT
TCCGGCGACCTGGTTCCACTGTCCTACATCGCTGGTCTGCTGACTGGTCGTCCGAACTCTAAAGCTGTTGGTCCGAC
TGGTGTTATCCTGTCCCCGGAAGAAGCGTTCAAACTGGCTGGTGTGGAAGGTGGTTTCTTTGAACTGCAACCGAA
AGAGGGCCTGGCACTGGTTAACGGTACCGCTGTTGGTTCTGGTATGGCGTCCATGGTTCTGTTCGAAGCTAACATC
CTGGCTGTTCTGGCGGAAGTGATGTCTGCTATCTTCGCTGAAGTTATGCAGGGTAAACCAGAGTTCACCGACCACC
TGACTCACAAACTGAAACACCACCCGGGTCAGATCGAAGCTGCTGCTATCATGGAACACATCCTGGACGGTTCTGC
CTACGTTAAAGCTGCTCAGAAACTGCACGAAATGGACCCGCTGCAAAAACCGAAACAGGACCGTTATGCTCTGCG
TACCTCTCCACAGTGGCTGGGCCCGCAAATCGAAGTTATCCGCTCCTCTACCAAGATGATCGAACGTGAAATCAAC
TCTGTTAACGACAACCCGCTGATCGACGTTTCCCGCAACAAAGCTATCCACGGTGGTAACTTCCAGGGGACCCCGA
TCGGCGTTTCCATGGACAACACCCGTCTGGCTATCGCAGCTATCGGTAAACTGATGTTCGCTCAATTCTCTGAACTG
GTTAACGACTTCTACAACAACGGTCTGCCATCTAACCTGTCTGGTGGTCGTAACCCGTCCCTGGACTATGGTTTCAA
AGGTGCAGAAATCGCTATGGCTTCCTACTGTTCTGAACTGCAATTCCTGGCTAACCCGGTTACCAACCACGTTCAGT
CCGCAGAACAGCACAACCAAGACGTTAACTCTCTGGGTCTGATCTCTTCTCGTAAAACCTCTGAAGCTGTTGAAAT
CCTGAAACTGATGTCCACTACCTTCCTGGTTGGTCTGTGTCAAGCTATCGACCTGCGTCACCTGGAAGAAAACCTG
AAATCCACCGTTAAAAACACCGTGTCTTCCGTGGCTAAACGTGTTCTGACGATGGGTGTTAATGGAGAACTGCACC
CGTCCCGTTTCTGCGAAAAAGACCTGCTGCGTGTTGTCGACCGTGAATACATCTTTGCTTACATCGACGACCCGTGC
TCCGCTACCTACCCACTGATGCAGAAACTGCGTCAGACCCTGGTTGAACATGCTCTGAAAAACGGTGACAACGAAC
GTAACCTGTCTACCTCCATCTTCCAGAAAATTGCAACCTTCGAAGATGAACTGAAAGCTCTGCTGCCGAAAGAAGT
TGAATCCGCTCGTGCAGCACTGGAATCTGGTAACCCTGCTATCCCAAACCGTATCGAAGAATGCCGTTCCTACCCG
CTGTACAAATTCGTTCGTAAAGAACTGGGCACTGAATACCTGACCGGTGAAAAAGTTACCTCCCCAGGTGAAGAG

TTCGAAAAAGTTTTCATCGCTATGTCCAAAGGTGAAATCATCGACCCGCTGCTGGAATGCCTGGAATCCTGGAATG GTGCTCCGCTGCCGATCTGC CATCACCATCACCACCACTAA

General Methods for Molecular Cloning, Transformation, and Engineering of Vectors

In general, most of the molecular cloning was performed by using the SLiCE method[4] (summarized below) and the mutagenesis was performed by using the Q5 mutagenesis method with KLD treatment or Golden Gate Mutagenesis[5] (see specific sections for details). For the SLiCE method, the SLiCE extract of *E. coli* TOP10 strain was prepared by the following protocol. The *E. coli* TOP10 cells were grown in 2× YT medium (16 g l^{-1} tryptone, 10 g l^{-1} yeast extract, 5 g l^{-1} NaCl) until OD_{600} reached 3.0. Then, the cells were harvested (5000 g, 10 min) and washed with ice-cold milli-Q water (5000 g, 15 min). The washed cell pellet was weighed and resuspended in CelLytic B Cell lysis reagent (Sigma, 0.3 ml per 0.25 g of pellet). It was lysed at room temperature for 10 min. Then, the clarified lysates (16000 g, 5 min) were mixed with glycerol (1:1, v/v) and stored at -80 ºC. The SLiCE buffer (10×) was prepared by dissolving $MgCl_2$ (0.1 M), ATP (10 mM), and DTT (10 mM) in Tris-HCl buffer (0.5 M, pH 7.5), and stored at -20 ºC. The SLiCE reaction (total volume 5 µl) was performed with two (or more) purified DNA fragments with 20 bp overlapping sequences (4 µl), SLiCE buffer (10×, 0.5 µl), and SLiCE extract (0.5 µl) at 37 ºC for 1 h. The SLiCE product was introduced into competent cells of *E. coli* by heat shock.

All PCRs were performed with Q5 DNA polymerase (New England Biolabs) according to the instruction. Purification of DNA fragments was done with a Gel Extraction Kit (Macherey-Nagel) according to the instruction. The sequences of primers are provided in Supplementary Table 2.

Transformation methods. The standard heat-shock transformation was used for most of the cloning works and for building small libraries (e.g., for the first 2 rounds of Q5 mutagenesis of PcPAL). Large libraries were introduced into *E. coli* cells by electroporation with an electroporation cuvette (1 mm gap) and default *E. coli* settings (1.8 kV) for the MicroPulser Electroporator (Biorad). Immediately after the electroporation shock, prewarmed LB medium (2 ml, 37 ºC) was added to the cuvette and the cells were cultured in an incubator for 2 h at 30 ºC, 200 rpm. A small portion of the cells (20 µl) was mixed with fresh LB medium (980 µl) and one tenth of the mixture (100 µl, corresponds to 1/1000 of the whole electroporation) was spread on an LB agar plate to test the electroporation efficiency. Half of the cells were added into LB medium (5 ml) containing kanamycin (50 mg l^{-1}) for isolation of the plasmids from the library. The other half of the cells were used for growth selection.

Genetic engineering of the vectors ETs/m/w/vw. The vectors ETs/m/w/vw were constructed by amplifying the plasmid backbone of pRSFduet-1 (Novagen) by using primers pRSF1623-R and pRSF2637-F and amplifying the pBR322ori of pETduet-1 (Novagen) by using primers pET2022-F and pET3983-R.

These two DNA fragments were purified and assembled by the SLiCE method to get an intermediate plasmid with pBR322ori, a kanamycin-resistant gene, and the expressing cassette of pRSFduet-1. To get the ETs vector, the strong constitutive promoter J23100 was introduced by amplifying the intermediate plasmid by using primers J23100S-F and J23100S-R (which replaced the 1st T7 promoter with J23100), and the PCR product was treated with the KLD Enzyme mix (New England Biolabs) and introduced into *E. coli* cells. Similarly, ETm/w/vw vectors were engineered by PCR with J23106M-F/J23106M-R, J23116W-F/J23116W-R, and J23109VW-F/J23109VW-R, respectively, followed by KLD treatment and transformation.

Genetic Engineering of AtTA

Genetic engineering of ETs/m/w/vw-AtTA. The AtTA gene was amplified from pGASTON-AtTA[1] by using primers AtTAopt-F and AtTA-R. The vectors ETs/m/w/vw were amplified by using the primers Duet-TT1stATG-R and Duet-XhoI-F. The purified AtTA fragment was assembled with the purified vector fragments by the SLiCE method to get the plasmids ETs/m/w/vw-AtTA.

Genetic construction of AtTA libraries. To construct the ETs-AtTA-Lib1 (H55-Y60-V62), the plasmid ETs-AtTA was amplified by using primers AtTA-6062X-F and AtTA-55X-R, and the PCR product was treated with the KLD Enzyme mix according to the instructions, purified and then introduced into the electrocompetent cells of *E. coli* BL21(DE3) by electroporation. The efficiency of electroporation was checked to ensure a full coverage of the library size. Half of the resulting cells were cultured for preparation of the plasmids of the library (its quality was checked by sequencing), and another half of the cells were subjected to growth selection (see Methods in the main text). The other AtTA libraries were constructed similarly: Lib2 (F115-E117) using primers AtTA-117X-F and AtTA-115X-R, Lib3 (V147-V149) using primers AtTA-149X-F and AtTA-147X-R, Lib4 (L182-W184-L187) using primers AtTA-1847X-F and AtTA-182X-R, Lib5 (T274-T275-A276) using primers AtTA-2756X-F and AtTA-274X-R.

Subcloning of AtTA variants. The genes encoding the AtTA variants (such as YHC) were subcloned to ETw and ETvw by the following procedures: the gene of AtTA(YHC) was amplified by using the primers Duet-TT1stATG-F and Duet-XhoI-R, and the targeted vector ETw was amplified by using primers Duet-TT1stATG-R and Duet-XhoI-F. The purified AtTA(YHC) fragment was assembled with the purified vector fragment by the SLiCE method to get the plasmid ETw-AtTA(YHC). Other subcloning of AtTA variants was performed similarly.

Genetic Engineering of CHAO

Genetic engineering of ETs/m/w/vw-CHAO. The gene encoding CHAO was amplified from the synthesized gene fragment by using the primers CHAO-F and CHAO-R. The vectors ETs/m/w/vw were amplified by using the primers Duet-1stATG-R and Duet-XhoI-F. The CHAO fragment was assembled with the vector fragments by the SLiCE method to get the plasmids ETs/m/w/vw-CHAO.

Genetic construction of CHAO libraries by Golden Gate Mutagenesis. The procedure of Golden Gate Mutagenesis follows a previous report[5] with some modifications. The BsaI restriction site inside the vectors ETm/w/vw-CHAO was first removed (termed domestication) by amplification of the plasmids by using the primers ET-dBsaI-F and ET-dBsaI-R. The PCR products were purified and subjected to a 40-cycle of digestion by BsaI-HFv2 (37 ºC, 2 min) and ligation by T4 ligase (20 ºC, 5 min) in a 15-μl system of T4 ligase buffer containing BsaI-HFv2 (0.5 μl) and T4 ligase (1 μl). The mixture was subjected to enzyme inactivation at 80 ºC for 20 min and then introduced into the competent cells of *E. coli* BL21(DE3) to get the vector ETm/w/vw-CHAO without BsaI restriction site. These plasmids were used as the templates to construct the CHAO libraries. To construct the ETm-CHAO-Lib1 (F88-F351-L353-F368), three DNA fragments were amplified from ETm-CHAO by using three pairs of primers, CHAO-88-F & CHAO-3513-R (fragment 1), CHAO-3513-F & CHAO-368-R (fragment 2), and CHAO-368-F & CHAO-88-R (fragment 3). The three fragments were purified and subjected to a 100-cycle of digestion by BsaI-HFv2 (37 ºC, 2 min) and ligation by T4 ligase (20 ºC, 5 min) in a 20-μl system of T4 ligase buffer containing BsaI-HFv2 (0.5 μl) and T4 ligase (1 μl). The mixture was subjected to enzyme inactivation at 80 ºC for 20 min and then introduced into the electrocompetent cells of *E. coli* BL21(DE3) SCm-KatE by electroporation. The efficiency of electroporation was checked to ensure a full coverage of the library size. Half of the resulting cells were cultured for preparation of the plasmids of the library (its quality was checked by sequencing), and another half of the cells were subjected to growth selection (see Methods in the main text). The other CHAO library was constructed similarly: Lib2 (T198-L199-M226-Y321) using primers CHAO-1989-F & CHAO-226-R (fragment 1), CHAO-226-F & CHAO-321-R (fragment 2), and CHAO-321-F & CHAO-1989-R (fragment 3).

Subcloning of CHAO variants. The genes of CHAO variants (such as MVLM) were subcloned to pRSFduet-1 by the following procedures: the gene encoding CHAO(MVLM) was amplified by using the primers Duet-1stATG-F and Duet-XhoI-R, and the targeted vector pRSFduet-1 was amplified by using the primers Duet-1stATG-R and Duet-XhoI-F. The purified CHAO(MVLM) fragment was assembled with the purified vector fragment by the SLiCE method to get the plasmid pRSF-CHAO(MVLM). Other subcloning of CHAO variants was performed similarly.

Genetic Engineering of PcPAL

Genetic engineering of ETs/m/w-PcPAL. The gene encoding PcPAL was amplified from the PcPAL plasmid[3] by using the primers PcPAL-F and PcPAL-His-R. The vectors ETs/m/w were amplified by using the primers Duet-1stATG-R and Duet-His-XhoI-F. The purified PcPAL fragment was assembled with the purified vector fragments by the SLiCE method to get the plasmids ETs/m/w-PcPAL. A previous study[6] showed that the C704S and C716S mutations increased the stability of PcPAL, thus C704S/C716S were introduced by Q5 mutagenesis using the primers PcPAL-704S-R and PcPAL-716S-F. The resulting ETs/m/w-PcPAL(C704S/C716S) were used for all the following studies.

Genetic construction of PcPAL libraries. The ETs-PcPAL-Lib (L134-F137-L138-L256-V259-I460) was constructed by sequentially performed Q5 mutagenesis rounds. The I460 was firstly introduced by amplifying the plasmid ETs-PcPAL using the primers PcPAL-460-F and PcPAL-460-R, and the PCR product was treated with the KLD Enzyme mix and then introduced into the competent cells of *E. coli* BL21(DE3). Using the resulting library ETs-PcPAL(I460) as the template, L256-V259 was then introduced by Q5 mutagenesis using the primers PcPAL-259-F and PcPAL-256-R. The efficiency of the heat-shock transformation was checked to ensure a full coverage of the library size. The resulting library of ETs-PcPAL(L256-V259-I460) was used as the template to further incorporate L134-F137-L138 by Q5 mutagenesis using the primers PcPAL-1378-F and PcPAL-134-R. The resulted library was introduced into the electrocompetent cells of *E. coli* BL21(DE3) Δ*tyrB* SCm-AroP by electroporation. The efficiency of electroporation was checked was checked to ensure a full coverage of the library size. Half of the resulting cells were cultured for preparation of the plasmids of the library (its quality was checked by sequencing), and another half of the cells were subjected to growth selection (see Methods in the main text). The other PcPAL libraries were constructed similarly.

Subcloning of PcPAL variants. The genes of PcPAL variants (such as AI) were subcloned to pRSFduet-1 by the following procedures: the gene of PcPAL(AI) was amplified by using the primers Duet-1stATG-F and Duet-XhoI-R, and the targeted vector pRSFduet-1 was amplified by using the primers Duet-1stATG-R and Duet-XhoI-F. The purified PcPAL(AI) fragment was assembled with the purified vector fragment by the SLiCE method to get the plasmid pRSF-PcPAL(AI). Other subcloning of PcPAL variants was performed similarly.

Genetic Engineering of Assisting Plasmids

The assisting plasmid SCm-KatE (expressing *E. coli* catalase) was constructed by the following procedures. The KatE gene was amplified from the genome of *E. coli* BL21(DE3) by using primers EcKatE-F and EcKatE-R. The vector SCm (with SC101ori, ampicillin resistant gene, and the constitutive promoter with medium strength) was amplified by using the primers Duet-1stATG-R and Duet-XhoI-F. The purified KatE fragment was assembled with the purified vector fragment by the SLiCE method to get the plasmid SCm-KatE.

The assisting plasmid SCm-AroP (expressing *E. coli* aromatic amino acid transporter) was constructed by the following procedures. The AroP gene was amplified from the genome of *E. coli* BL21(DE3) by using the primers EcAroP-F and EcAroP-R. The vector SCm (with SC101ori, ampicillin-resistant gene, and the constitutive promoter with medium strength) was amplified by using the primers Duet-1stATG-R and Duet-XhoI-F. The purified AroP fragment was assembled with the purified vector fragment by the SLiCE method to get the plasmid SCm-AroP.

Genome Engineering of *E. coli* BL21(DE3) Δ*tyrB*

The *E. coli* BL21(DE3)Δ*tyrB* strain was engineered following a previously reported CRISPR-Cas9 method[7] with two key plasmids pCas (Addgene #62225) and pTargetF (Addgene #62226). pTarget-dTryB plasmid was engineered by first engineering the flanking sequences of *tyrB* gene by assembling three DNA fragments: tyrB-Up fragment (amplified from *E. coli* genome by using the primers tyrB-Up-F and tyrB-Up-R), tyrB-Down fragment (amplified from *E. coli* genome by using the primers tyrB-Down-F and tyrB-Down-R), and pTarget fragment (amplified by using the primers pTarget-R and pTarget-F). Then, the gRNA was introduced by using Q5 mutagenesis with the primers dTyrB-gRNA-F and dTyrB-gRNA-R. The resulting pTarget-dTyrB plasmid was sequenced to confirm the existence of gRNA and flanking sequences of *tyrB* gene. *E. coli* BL21(DE3)Δ*tyrB* strain was engineered by using pCas and pTarget-dTyrB according to the previously reported procedure,[7] briefly outlined: 1) pCas was introduced into the *E. coli* BL21(DE3) cells, 2) electrocompetent cells of *E. coli* BL21(DE3) pCas were prepared with an arabinose-induced λ-Red system, 3) pTarget-dTyrB was introduced by electroporation, 4) selection was performed on LB plates with kanamycin and streptomycin, 5) perform colony PCR was performed for genotyping the colonies, 6) the plasmid pTarget-dTyrB (by using IPTG) and pCas (by culturing at 37 °C) were sequentially cured. The resulting *E. coli* BL21(DE3)*ΔtyrB* strain was confirmed by PCR amplification and sequencing of the genome sequence flanking the *tyrB* gene.

Expression of Enzymes and Preparation of Whole-cell Catalysts

The plasmids (ETs/ETm/pRSFduet-1) containing the genes encoding the AtTA/CHAO/PcPAL variants were transformed into *E. coli* BL21(DE3) cells, and the resulting cells were incubated in a culture tube containing LB medium (5 ml) with kanamycin (50 mg ml^{-1}) at 37 ºC, 180 rpm for 6-8 h. The culture was transferred into a flask (500 ml) containing TB medium (100 ml) with kanamycin (50 mg ml^{-1}) and incubated at 37 ºC, 180 rpm. When the OD_{600} of the culture reached 0.6, the culture was incubated at 20 ºC (AtTA) or 22 ºC (CHAO and PcPAL), 180 rpm for another 12-14 h. For the pRSFduet-1 plasmid, IPTG (0.5 mM, isopropyl β-D-thiogalactopyranoside) was added (at OD_{600} = 0.6) to induce the expression of enzymes. For the ETs or ETm plasmid, no IPTG is needed. The cells were harvested by centrifugation (4000 g, 20 min).

Purification of Enzymes

For purification of AtTA variants, the cell pellets were resuspended in HEPES buffer (50 mM, pH 7.5) containing NaCl (300 mM), PLP (0.1 mM), imidazole (10 mM), and then lysed by ultrasonication (50% power, 50% pulse, 5 min for two times). The lysate was clarified by centrifugation (10000 g, 4 ºC, 1 h) and purified by affinity chromatography (Ni-NTA agarose) with the following buffers: washing buffer containing HEPES (50 mM, pH 7.5), PLP (0.1 mM), NaCl (300 mM), and imidazole (20 mM), and elution buffer containing HEPES (50 mM, pH 7.5), PLP (0.1 mM), NaCl (300 mM), and imidazole (300 mM). The AtTA variants were desalted in HEPES buffer (50 mM pH 7.5) with PLP (0.1 mM) using the PD-10 desalting column (GE Healthcare). The purified AtTA variants were stored in 30% glycerol at -20 ºC.

For purification of CHAO and PcPAL variants, the same procedure was employed with some difference in the buffer: sodium phosphate buffer (50 mM, pH 7.5) without PLP was used instead of HEPES buffer (50 mM, pH 7.5) with PLP (0.1 mM).

For purification of AtTA(RHC) for crystallization, the cells were resuspended in lysis buffer containing HEPES (25 mM, pH 7.0), NaCl (150 mM), PLP (0.1 mM), imidazole (20 mM), then disrupted by a French Press, cell debris was removed by centrifugation (17000 g, 4 ºC, 1 h). The supernatant was then applied onto a Ni-NTA column FPLC system (GE Health care) equilibrated with buffer (20 mM HEPES, pH 7.0, 150 mM NaCl, 0.1 mM PLP, 20 mM imidazole), target proteins were eluted at ~100 mM imidazole. Proteins were concentrated and applied to a size exclusion column (Hiload superdex 200 16/600, 120 ml, GE Healthcare) equilibrated with buffer (20 mM HEPES, pH 7.0, 0.1 mM PLP, 150 mM NaCl) at a flow rate of 1 ml min^{-1}. The purity of each purification step was checked by SDS-PAGE gel. The purified proteins

were concentrated in buffer (20 mM HEPES, pH 7.0, 0.1 mM PLP, 150 mM NaCl) for crystallization screening.

Activity Assays

The specific activities of purified AtTA variants were determined by the D-amino acid oxidase assay reported previously. The reaction was performed in microtiter plates (200 µl assay volume) with (*R*)-**1** (2.5 mM) as amine donor and pyruvate (2.5 mM) as amine acceptor, D-amino acid oxidase (0.7 U ml^{-1}) from *T. variabilis* (TvDAAO), horseradish peroxidase (HRP, 22 U ml^{-1}), Ampliflu Red (50 µM) in CHES buffer (50 mM, pH 9.0), and an appropriate amount of AtTA variants (10 µl of 1-1000× dilution). The formation of Resorufin was quantified by following the increase of absorption at 560 nm over time by a Tecan infinite 200pro plate reader.

The specific activities of purified CHAO variants were determined by the Ampliflu Red assay. The reaction was performed in microtiter plates (200 µl assay volume) with substrate (5 mM), horseradish peroxidase (HRP, 2 U per well), Ampliflu Red (50 µM) in potassium phosphate buffer (50 mM, pH 7.0), and an appropriate amount of CHAO (10 µl of 1-1000× dilution). The formation of Resorufin was quantified by following the increase of absorption at 560 nm over time by a Tecan infinite 200pro plate reader.

The specific activities of purified PcPAL variants were determined by measuring the absorbance of **6** at 330 nm. The amination reaction (**6**→**5**) was performed in microtiter plates (200 µl assay volume) with **6** (0.5 mM) in NH_3/NH_4Cl buffer (6 M, pH 9.5), and an appropriate amount of PcPAL (20 µl). The deamination reaction (**5**→**6**) was performed in microtiter plates (200 µl assay volume) with (*S*)-**5** (1 mM) in potassium phosphate buffer (10 mM, pH 7.0), and an appropriate amount of PcPAL (10 µl). The decrease/formation of **6** was quantified by following the change of absorption at 330 nm over time by a Tecan infinite 200pro plate reader.

Analytical Methods

Analysis of **1** and **2** was performed using a Hitachi Chromaster HPLC system with a Luna Omega 5 µm PS C18 100A LC column (150 mm × 4.6 mm) and the following program: 20% acetonitrile and 80% water (contains 0.1% TEA) at a constant flow rate (1.0 ml min^{-1}) and the absorbance was detected at 210 nm. Chromaster System Manager was used to collect the data. Retention times: 3.4 min for **1**, 12.6 min for **2**. The compounds were quantified based on the peak areas (absorbance at 210 nm, external standard).

The *ee* of **1** was determined by chiral GC analysis using a Shimadzu GC-2010 Plus system with an FID detector. The samples of **1** (in CH_2Cl_2) were firstly derivatized to the trifluoroacetamide by adding a 20-fold excess of trifluoroacetic anhydride. The excess anhydride and residual trifluoroacetic acid were removed by nitrogen purging, and the derivatized compound was dissolved in CH_2Cl_2 and analyzed by the following program. Column: heptakis-(2,3-di-*O*-acetyl-6-*O*-*tert*-butyldimethylsilyl)-cyclodextrin (25 m × 0.25 mm). Temperature program: start at 160 °C for 10.5 min, then increase to 180 °C at 10 °C min^{-1}, hold at 180 °C for 20 min. Shimadzu LabSolutions was used to collect the data. Retention times: 12.5 min for the trifluoroacetamide of (*S*)-**1**, 13.1 min for the trifluoroacetamide of (*R*)-**1**.

The concentration and *ee* of **3** were simultaneously determined by chiral HPLC analysis on an Agilent 1200 infinity system with a Chiralcel OD-3 column (3 µm, 4.6 mm × 250 mm, Daicel) and the following program: 80% *n*-hexane and 20% isopropanol at a constant flow rate (1.0 ml min^{-1}) and the absorbance was detected at 210 nm. Agilent OpenLab was used to collect the data. Retention times: 3.5 min for internal standard (ethylbenzene), 7.2 min for (*S*)-**3**, and 8.0 min for (*R*)-**3**. The compounds were quantified based on the peak areas (absorbance at 210 nm) using the internal standard.

The concentration of **5** was determined by HPLC analysis on an EasySep®-3030 HPLC system (Unimicro Technologies, Shanghai) with an Agilent Poroshell 120 SB-C18 column (2.7 µm, 4.6 mm × 100 mm) and the following program: 40% H_2O (contains 0.1% trifluoracetic acid) and 60% methanol at a constant flow rate (0.5 ml min^{-1}) and the absorbance was detected at 210 nm. Unicmicro ChromStation was used to collect the data. Retention times: 3.5 min for **5**, 4.5 min for internal standard (acetophenone), and 12.0 min for **6**. The compounds were quantified based on the peak areas (absorbance at 210 nm) using the internal standard.

The *ee* of **5** was determined by chiral HPLC analysis on a Wooking K2025 HPLC system with a Crownpak CR(+) column (5 µm, 4.6 mm × 150 mm, Daicel) and the following program: 85% H_2O (contains 0.1% trifluoracetic acid) and 15% methanol at a constant flow rate (1.0 ml min^{-1}) and the absorbance was detected at 210 nm. WookingLab was used to collect the data. Retention times: 28.1 min for (*R*)-**5**, and 36.4 min for (*S*)-**5**.

Crystallization, Data Collection, Structure Determination of AtTA(RHC)

All crystallization experiments were conducted at 25°C using the sitting-drop vapor-diffusion method. In general, 1 µl AtTA(RHC) (20 mg ml^{-1} in HEPES buffer (20 mM, pH 7.0, with 0.1 mM PLP, 150 mM NaCl)) was mixed with 1 µl of reservoir solution in 48-well Cryschem Plates, then equilibrated against 100 µl of

the reservoir at 25 °C. The crystals of AtTA(RHC) were obtained under the following crystallization condition: 0.1M HEPES pH 7.5, 10% PEG8000, 15% ethylglycerol. Within one week, the crystals reached dimensions suitable for X-ray diffraction. 0.1 mM PLP + 1 mM (*R*)-**1** were used in co-crystallization and soaking experiments.

All of the X-ray diffraction data were tested and collected at the Beamline 14.1 at BESSY. The crystals were mounted in a cryoloop, soaked with cryoprotectant solution (0.1M HEPES pH 7.5, 10% PEG8000, 20% ethylglycerol) prior to data collection at 100 K. The diffraction images were processed by using XDS.[8] Both of the crystal structures were solved by molecular replacement (MR) method with Phaser[9] from the Phenix[10] suite using the structure of AtTA(wt) (PDB code 4CE5)[11] as the search model. The further refinement was carried out using programs phenix.refine[12] and medel building with Coot.[13] Prior to structural refinements, 5% randomly selected reflections were set aside for calculating R_{free} as a monitor. Data collection and refinement statistics are summarized in Supplementary Table 1. All figures were prepared by using the PyMOL program (https://pymol.org/2/).

Homology Modeling and Docking Experiment

If not stated otherwise, the modeling and docking experiment were mainly performed with YASARA Structure version 20.4.24.

For the *in silico* study of AtTA(RHC), the PLP-(*R*)-**1** complex (external aldimine) was created with ChemDraw and underwent energy minimization (force field: Yasara2) and the bonds and chirality of the complex were double-checked. The existing PLP and (*R*)-**1** (partial density) in the crystal structure of AtTA(RHC) (PDB: 7XG5) was removed to prepare the structure for docking. The complex was docked into the active site of AtTA(RHC) using the AutoDock VINA implemented in YASARA Structure (force field: AMBER03). The VINA docking runs generated 25 poses (5 distinct complex conformations). The pose with the lowest binding energy shows a very similar binding of the PLP part compared to the binding of PLP in the AtTA(wt) and exhibits a hydrogen bond between the guanidinium group of H55R and the carbonyl group of (*R*)-**1**. Thus, this pose was used to create the figure with the PyMOL program.

For the *in silico* study of CHAO(MVLM), the homology model of CHAO(MVLM) was built using the structure of CHAO(wt) (PDB: 4I59)[14] as the template by YASARA. The model was refined to the optimal conformation by performing a 500 ps MD simulation. The snapshot with the lowest energy was used as the structure for docking. (*S*)-**3** was created with ChemDraw and underwent energy minimization (force field: Yasara2) and the bonds and chirality were double-checked. (*S*)-**3** was docked into the active

site of CHAO(MVLM) using the AutoDock VINA implemented in YASARA Structure (force field: AMBER03). (*S*)-**3** was docked 32 times with VINA against each of the 5 receptors in the ensemble, yielding the 160 results. A pose was found in the results with a suitable distance (3-4 Å) between the C-atom α to the amine of (*S*)-**3** and the N5 of the flavin. The pose was used to create the figure with the PyMOL program.

For the *in silico* study of PcPAL(AI), the homology model of PcPAL(AI) was built using the structure of PcPAL(wt) (PDB: 6F6T)[15] as the template by SWISS-MODEL. The GMQE score of the model was 0.89 and the QMEANDisCo Global score is 0.85 ± 0.05. **6** was created with ChemDraw and underwent energy minimization (force field: Yasara2), and the atoms and bonds were double-checked. **6** was docked into the active site of PcPAL(AI) using the AutoDock VINA implemented in YASARA Structure (force field: AMBER03). The VINA docking runs generated 25 poses (3 distinct complex conformations). The pose with the lowest binding energy shows a very similar binding of **6** compared to the binding of (*R*)-(1-amino-2-phenylethyl)phosphonic acid (inhibitor) in the PcPAL(wt). Thus, this pose was used to create the figure with the PyMOL program.

Preparative Scale Syntheses

The preparation of (*R*)-**1** followed the same procedure as the Methods of the main text. (*R*)-**1** was isolated as yellow oil (1.96 g, 98% yield, 98% *ee*). ^{1}H NMR (300 MHz, DMSO-d_6) δ: 3.83–3.79 (d, J = 10.5 Hz, 1H), 3.76–3.69 (m, 1H), 2.69–2.62 (t, J = 11.5 Hz, 1H), 2.54–2.47 (m, 1H), 2.39 (br, 1H), 1.81–1.75 (m, 1H), 1.61–1.55 (m, 1H), 1.39 (s, 9H), 2.07 (brs, 2H), 1.32–1.26 (m, 1H), 1.11–1.07 (m, 1H).

For the preparative deracemization to produce (*R*)-**3** fresh *E. coli* cells containing pRSF-CHAO(MVLM) and SCm-KatE were employed as whole-cell catalysts. The cell pellets of a 200-ml culture were first washed with Tris-HCl buffer (100 mM, pH 8.0) and subjected to centrifugation again (4000 g, 15 min). The supernatant was discarded and the cell pellets were resuspended in new Tris-HCl buffer (100 mM, pH 8.0) and the optical density of the cell suspensions was measured. To an Erlenmeyer flask (1000 ml), the cell suspensions, a stock solution of *rac*-**3** (428 mg, 500 mM HCl salt in water), a stock solution of NH_3-BH_3 (1 M in Tris-HCl buffer), Tris-HCl buffer (100 mM, pH 8.0), and *n*-dodecane (100 ml) were added to form a two-phase catalytic system composed of the aqueous buffer (100 ml) containing *rac*-**3** (25 mM), cells (10 g dcw l^{-1}) and NH_3-BH_3 (500 mM) and organic phase (100 ml). The flask was incubated at 30 ^{o}C, 200 rpm for 4 h. A sample of the reaction (100 μl) was taken, mixed with NaOH solution (5 M, 100 μl), and extracted with *n*-hexane (1 ml) to check the *ee* by chiral HPLC analysis. Then, a new batch of cell suspensions (~10 ml, from 200-ml culture) was added to the reaction mixture, and continued reaction for another 4 h (8 h in total). The *ee* reached > 99% by chiral HPLC analysis. The reaction was quenched by

adding HCl solution to reach pH 1. The mixture was centrifuged (4000 g, 5 min) to separate the organic phase (*n*-dodecane), and then the aqueous phase was extracted by *n*-hexane (50 ml) three times. The aqueous phase was basified by NaOH solution to pH 13, and extracted with *n*-hexane (100 ml) five times. The organic phase was combined and dried with Na_2SO_4. Further evaporation and drying under vacuum overnight offered (*R*)-**3** as yellowish oil (295 mg, 69% yield, > 99% *ee*). ^{1}H NMR (600 MHz, $CDCl_3$) δ: 8.15–8.13 (d, *J* = 8.4 Hz, 1H), 7.88–7.87 (d, *J* = 8.1 Hz, 1H), 7.76–7.75 (d, *J* = 8.1 Hz, 1H), 7.65–7.64 (d, *J* = 7.2 Hz, 1H), 7.54–7.47 (m, 3H), 4.97–4.94 (q, *J* = 6.8 Hz, 1H), 1.85 (br, 2H), 1.55–1.54 (d, *J* = 6.6 Hz, 3H). The NMR data are consistent with those in a previous report.[16]

Supplementary References

1. Höhne, M., Schätzle, S., Jochens, H., Robins, K. & Bornscheuer, U. T. Rational assignment of key motifs for function guides in silico enzyme identification. *Nat. Chem. Biol.* **6**, 807–813 (2010).
2. Li, G. et al. Substrate profiling of cyclohexylamine oxidase and its mutants reveals new biocatalytic potential in deracemization of racemic amines. *Appl. Microbiol. Biotechnol.* **98**, 1681–1689 (2014).
3. Bartsch, S. & Bornscheuer, U. T. A single residue influences the reaction mechanism of ammonia lyases and mutases. *Angew. Chem. Int. Ed.* **48**, 3362–3365 (2009).
4. Zhang, Y., Werling, U. & Edelmann, W. SLiCE: a novel bacterial cell extract-based DNA cloning method. *Nucleic Acids Res.* **40**, e55 (2012).
5. Püllmann, P. et al. Golden mutagenesis: an efficient multi-site-saturation mutagenesis approach by Golden Gate cloning with automated primer design. *Sci. Rep.* **9**, 10932 (2019).
6. Nagy, E. M. Z. et al. Mapping the hydrophobic substrate binding site of phenylalanine ammonia-lyase from *Petroselinum crispum*. *ACS Catal.* **9**, 8825–8834 (2019).
7. Jiang, Y. et al. Multigene editing in the *Escherichia coli* genome via the CRISPR-Cas9 system. *Appl. Environ. Microbiol.* **82**, 3693 (2016).
8. Kabsch, W. Xds. *Acta Crystallogr. D Biol. Crystallogr.* **66**, 125–132 (2010).
9. McCoy, A. J. et al. Phaser crystallographic software. *J. Appl. Crystallogr.* **40**, 658–674 (2007).
10. Liebschner, D. et al. Macromolecular structure determination using X-rays, neutrons and electrons: recent developments in Phenix. *Acta Crystallogr. D Struct. Biol.* **75**, 861–877 (2019).
11. Łyskowski, A. et al. Crystal structure of an (*R*)-selective ω-transaminase from *Aspergillus terreus*. *PLoS ONE* **9**, e87350 (2014).
12. Afonine, P. V. et al. Towards automated crystallographic structure refinement with phenix.refine. *Acta crystallogr. D Biol. Crystallogr.* **68**, 352–367 (2012).

13. Emsley, P. & Cowtan, K. Coot: model-building tools for molecular graphics. *Acta crystallogr. D Biol. Crystallogr.* **60**, 2126–2132 (2004).
14. Mirza, I. A. et al. Structural analysis of a novel cyclohexylamine oxidase from *Brevibacterium oxydans* IH-35A. *PLoS ONE* **8**, e60072 (2013).
15. Bata, Z. et al. Substrate tunnel engineering aided by X-ray crystallography and functional dynamics swaps the function of MIO-enzymes. *ACS Catal.* **11**, 4538–4539 (2021).
16. Marx, L. et al. Chemoenzymatic approaches to the synthesis of the calcimimetic agent Cinacalcet employing transaminases and ketoreductases. *Adv. Synth. Catal.* **360**, 2157–2165 (2018).
