# Supplementary material for: A growth selection system for the directed evolution of amine-forming or converting enzymes
Source: Nat Commun. 2022 Dec 3;13:7458. doi: 10.1038/s41467-022-35228-y (PMC9718777; doi:10.1038/s41467-022-35228-y)
Supplement: Supplementary file 3 — Reporting Summary [file 41467_2022_35228_MOESM3_ESM.pdf]

nature portfolio

Corresponding author(s): Shuke Wu, Uwe Bornscheuer

Last updated by author(s): Nov 22, 2022

Reporting Summary

Nature Portfolio wishes to improve the reproducibility of the work that we publish. This form provides structure for consistency and transparency in reporting. For further information on Nature Portfolio policies, see our Editorial Policies and the Editorial Policy Checklist.

Statistics

For all statistical analyses, confirm that the following items are present in the figure legend, table legend, main text, or Methods section.

n/a	Confirmed	
☐	☒	The exact sample size (*n*) for each experimental group/condition, given as a discrete number and unit of measurement
☐	☒	A statement on whether measurements were taken from distinct samples or whether the same sample was measured repeatedly
☒	☐	The statistical test(s) used AND whether they are one- or two-sided<br>*Only common tests should be described solely by name; describe more complex techniques in the Methods section.*
☒	☐	A description of all covariates tested
☒	☐	A description of any assumptions or corrections, such as tests of normality and adjustment for multiple comparisons
☐	☒	A full description of the statistical parameters including central tendency (e.g. means) or other basic estimates (e.g. regression coefficient) AND variation (e.g. standard deviation) or associated estimates of uncertainty (e.g. confidence intervals)
☒	☐	For null hypothesis testing, the test statistic (e.g. *F*, *t*, *r*) with confidence intervals, effect sizes, degrees of freedom and *P* value noted<br>*Give P values as exact values whenever suitable.*
☒	☐	For Bayesian analysis, information on the choice of priors and Markov chain Monte Carlo settings
☒	☐	For hierarchical and complex designs, identification of the appropriate level for tests and full reporting of outcomes
☒	☐	Estimates of effect sizes (e.g. Cohen's *d*, Pearson's *r*), indicating how they were calculated

Our web collection on statistics for biologists contains articles on many of the points above.

Software and code

Policy information about availability of computer code

Data collection	Collection of OD, absorption and fluorescence data: Tecan I-control (version 2.0.10.0). Collection of HPLC data: Chromaster System Manager (version 7.21), Agilent OpenLab (version 2.18.18), Unicmicro ChromStation (version 2.2.0.0), WookingLab (version 00.03.51.02). Collection of GC data: Shimadzu LabSolutions (version 5.106). Collection of protein structure data: XDS (version Jan 31 2020), Phaser (version 2.8.3) from the Phenix suite. Modeling and docking data: YASARA Structure (version 20.4.24).
Data analysis	Microsoft Excel (version 2019) was used to draw charts. PyMOL (version 1.8.0.2) was used for preparing structure figures. GraphPad Prism (version 8.3.0) was used for analysis of enzyme kinetic data. Phenix.refine (version 1.20.1) and Coot (version 0.9.8) were used for refinement of crystal structure data. MestReNova (version 14.0.0) was used for analysis of NMR data. ChemDraw (version 19.0.0.22) was used to draw the schemes. Microsoft Power Point (version 2019) was used to prepare and combine figures.

For manuscripts utilizing custom algorithms or software that are central to the research but not yet described in published literature, software must be made available to editors and reviewers. We strongly encourage code deposition in a community repository (e.g. GitHub). See the Nature Portfolio guidelines for submitting code & software for further information.

Data

Policy information about availability of data

All manuscripts must include a data availability statement. This statement should provide the following information, where applicable:

- Accession codes, unique identifiers, or web links for publicly available datasets
- A description of any restrictions on data availability
- For clinical datasets or third party data, please ensure that the statement adheres to our policy

The structural data used in this study are from Protein Data Bank under accession codes 4CE5 [https://www.rcsb.org/structure/4CE5], 4I59 [https://www.rcsb.org/

structure/4I59], and 6HQF [https://www.rcsb.org/structure/6HQF]. The structural data generated in this study have been deposited in the Protein Data Bank under accession codes 7XG5 [https://www.rcsb.org/structure/7XG5] and 7XG6 [https://www.rcsb.org/structure/7XG6]. Additional data supporting the findings of this study are available as Supplementary Information. Source data are provided with this paper. All unique biological materials (plasmids and strains) are readily available from the corresponding authors upon request.

Field-specific reporting

Please select the one below that is the best fit for your research. If you are not sure, read the appropriate sections before making your selection.

- [x] Life sciences
- [ ] Behavioural & social sciences
- [ ] Ecological, evolutionary & environmental sciences

For a reference copy of the document with all sections, see nature.com/documents/nr-reporting-summary-flat.pdf

Life sciences study design

All studies must disclose on these points even when the disclosure is negative.

Sample size	No sample-size calculation was performed. A sample size of 3 was used for most of the experiments (measurements of reaction conversion, enzyme activity, and cell growth), because 3 samples are usually sufficient to calculate the means and standard deviations and allow to distinguish the performance of different enzyme mutants. A sample size of 1 was used for preparative synthesis of (R)-1 by different AtTA variants (Fig 3e, because of limited availability of automatic pH controlling apparatus and substrate).
Data exclusions	No data were exclusions from the study.
Replication	Most of measurements of reaction conversion, enzyme activity, and cell growth were performed in triplicate, and all the replication were successful. The preparative synthesis of (R)-1 by different AtTA variants was conduct once (Fig 3e) because of limited availability of automatic pH controlling apparatus and substrate.
Randomization	During the growth selection, randomly half of the cells after electroporation was used for preparation of the plasmid library, and the other half was used for growth selection. The mixed cells were treated equally (on the same plate or in the same tube) during the growth selection. Randomization was not applicable for other experiment.
Blinding	Blinding is not relevant to this study, because we need to know the identity of chemicals (for safety reasons).

Reporting for specific materials, systems and methods

We require information from authors about some types of materials, experimental systems and methods used in many studies. Here, indicate whether each material, system or method listed is relevant to your study. If you are not sure if a list item applies to your research, read the appropriate section before selecting a response.

Materials & experimental systems

n/a	Involved in the study	
[x]	[ ]	Antibodies
[x]	[ ]	Eukaryotic cell lines
[x]	[ ]	Palaeontology and archaeology
[x]	[ ]	Animals and other organisms
[x]	[ ]	Human research participants
[x]	[ ]	Clinical data
[x]	[ ]	Dual use research of concern

Methods

n/a	Involved in the study	
[x]	[ ]	ChIP-seq
[x]	[ ]	Flow cytometry
[x]	[ ]	MRI-based neuroimaging
